# OsRH52A, a DEAD-box protein, regulates functional megaspore specification and is required for embryo sac development in rice

**DOI:** 10.1093/jxb/erae180

**Published:** 2024-04-20

**Authors:** Jinghua Huang, Zhengping Qiao, Hang Yu, Zijun Lu, Weibin Chen, Junming Lu, Jinwen Wu, Yueming Bao, Muhammad Qasim Shahid, Xiangdong Liu

**Affiliations:** State Key Laboratory for Conservation and Utilization of Subtropical Agro-Bioresources, Guangdong Laboratory for Lingnan Modern Agriculture, South China Agricultural University, Guangzhou 510642, China; Guangdong Provincial Key Laboratory of Plant Molecular Breeding, South China Agricultural University, Guangzhou 510642, China; Guangdong Base Bank for Lingnan Rice Germplasm Resources, College of Agriculture, South China Agricultural University, Guangzhou 510642, China; State Key Laboratory for Conservation and Utilization of Subtropical Agro-Bioresources, Guangdong Laboratory for Lingnan Modern Agriculture, South China Agricultural University, Guangzhou 510642, China; Guangdong Provincial Key Laboratory of Plant Molecular Breeding, South China Agricultural University, Guangzhou 510642, China; Guangdong Base Bank for Lingnan Rice Germplasm Resources, College of Agriculture, South China Agricultural University, Guangzhou 510642, China; State Key Laboratory for Conservation and Utilization of Subtropical Agro-Bioresources, Guangdong Laboratory for Lingnan Modern Agriculture, South China Agricultural University, Guangzhou 510642, China; Guangdong Provincial Key Laboratory of Plant Molecular Breeding, South China Agricultural University, Guangzhou 510642, China; Guangdong Base Bank for Lingnan Rice Germplasm Resources, College of Agriculture, South China Agricultural University, Guangzhou 510642, China; State Key Laboratory for Conservation and Utilization of Subtropical Agro-Bioresources, Guangdong Laboratory for Lingnan Modern Agriculture, South China Agricultural University, Guangzhou 510642, China; Guangdong Provincial Key Laboratory of Plant Molecular Breeding, South China Agricultural University, Guangzhou 510642, China; Guangdong Base Bank for Lingnan Rice Germplasm Resources, College of Agriculture, South China Agricultural University, Guangzhou 510642, China; State Key Laboratory for Conservation and Utilization of Subtropical Agro-Bioresources, Guangdong Laboratory for Lingnan Modern Agriculture, South China Agricultural University, Guangzhou 510642, China; Guangdong Provincial Key Laboratory of Plant Molecular Breeding, South China Agricultural University, Guangzhou 510642, China; Guangdong Base Bank for Lingnan Rice Germplasm Resources, College of Agriculture, South China Agricultural University, Guangzhou 510642, China; State Key Laboratory for Conservation and Utilization of Subtropical Agro-Bioresources, Guangdong Laboratory for Lingnan Modern Agriculture, South China Agricultural University, Guangzhou 510642, China; Guangdong Provincial Key Laboratory of Plant Molecular Breeding, South China Agricultural University, Guangzhou 510642, China; Guangdong Base Bank for Lingnan Rice Germplasm Resources, College of Agriculture, South China Agricultural University, Guangzhou 510642, China; State Key Laboratory for Conservation and Utilization of Subtropical Agro-Bioresources, Guangdong Laboratory for Lingnan Modern Agriculture, South China Agricultural University, Guangzhou 510642, China; Guangdong Provincial Key Laboratory of Plant Molecular Breeding, South China Agricultural University, Guangzhou 510642, China; Guangdong Base Bank for Lingnan Rice Germplasm Resources, College of Agriculture, South China Agricultural University, Guangzhou 510642, China; State Key Laboratory for Conservation and Utilization of Subtropical Agro-Bioresources, Guangdong Laboratory for Lingnan Modern Agriculture, South China Agricultural University, Guangzhou 510642, China; Guangdong Provincial Key Laboratory of Plant Molecular Breeding, South China Agricultural University, Guangzhou 510642, China; Guangdong Base Bank for Lingnan Rice Germplasm Resources, College of Agriculture, South China Agricultural University, Guangzhou 510642, China; State Key Laboratory for Conservation and Utilization of Subtropical Agro-Bioresources, Guangdong Laboratory for Lingnan Modern Agriculture, South China Agricultural University, Guangzhou 510642, China; Guangdong Provincial Key Laboratory of Plant Molecular Breeding, South China Agricultural University, Guangzhou 510642, China; Guangdong Base Bank for Lingnan Rice Germplasm Resources, College of Agriculture, South China Agricultural University, Guangzhou 510642, China; State Key Laboratory for Conservation and Utilization of Subtropical Agro-Bioresources, Guangdong Laboratory for Lingnan Modern Agriculture, South China Agricultural University, Guangzhou 510642, China; Guangdong Provincial Key Laboratory of Plant Molecular Breeding, South China Agricultural University, Guangzhou 510642, China; Guangdong Base Bank for Lingnan Rice Germplasm Resources, College of Agriculture, South China Agricultural University, Guangzhou 510642, China; University Clermont Auvergne, France

**Keywords:** Cell specification, chalazal functional megaspore, DEAD-box RNA helicase, double-female-gametophyte, embryo sac, *Oryza sativa*, rice

## Abstract

The development of the embryo sac is an important factor that affects seed setting in rice. Numerous genes associated with embryo sac (ES) development have been identified in plants; however, the function of the DEAD-box RNA helicase family genes is poorly known in rice. Here, we characterized a rice DEAD-box protein, RH52A, which is localized in the nucleus and cytoplasm and highly expressed in the floral organs. The knockout mutant *rh52a* displayed partial ES sterility, including degeneration of the ES (21%) and the presence of a double-female-gametophyte (DFG) structure (11.8%). The DFG developed from two functional megaspores near the chalazal end in one ovule, and 3.4% of DFGs were able to fertilize via the sac near the micropylar pole in *rh52a*. RH52A was found to interact with MFS1 and ZIP4, both of which play a role in homologous recombination in rice meiosis. RNA-sequencing identified 234 down-regulated differentially expressed genes associated with reproductive development, including two, *MSP1* and *HSA1b*, required for female germline cell specification. Taken together, our study demonstrates that *RH52A* is essential for the development of the rice embryo sac and provides cytological details regarding the formation of DFGs.

## Teaser

*RH52A* is vital for female gametophyte development in rice, and its mutation results in the production of double-female-gametophytes that develop from two functional chalazal megaspores.

## Introduction

Rice (*Oryza sativa*) is one of the most important crops in the world. The seed-setting rate is the crucial element affecting grain yield in rice, and it depends on pollen and embryo sac fertility (Hou *et al.*, 2019). Correct embryo sac development is one of the essential factors for successful reproduction, and a comprehensive understanding of the molecular mechanisms involved is of great importance for the future advancement of female-sterile ‘restorer lines’ for mechanized seed production of hybrid rice (Mao *et al.*, 2021).

As a member of the *Gramineae*, rice typically exhibits development and differentiation of a single archesporial cell (AC), which gives rise to the megaspore mother cell, also known as the embryo-sac mother cell (EMC) (Nonomura *et al.*, 2003). Subsequently, the EMC undergoes meiosis, resulting in the formation of four megaspores. Among these, one megaspore in close proximity to the chalazal region successfully progresses into a functional megaspore (FM), while the remaining three undergo degeneration. The FM develops into a mono-nucleate embryo sac, which undergoes mitosis three times to form a bi-, tetra-, and eight-nucleate embryo sac, and a mature embryo sac (Liu *et al.*, 1997; Yang *et al.*, 2010; Wang *et al.*, 2021; Hu *et al.*, 2023). A mature embryo sac, also called a female gametophyte or megagametophyte, has an egg apparatus comprised of one egg cell and two synergids in the micropylar part, two polar nuclei, and a group of antipodal cells in the chalazal part (Liu *et al.*, 1997; Ao, 2013).

Numerous genes regulating the development of embryo sac during the AC, EMC, FM, and mitosis of FM stages have been identified. During the AC and EMC stages, *MULTIPLE SPOROCYTE1* (*MSP1*), *TAPETUM DETERMINANT1-LIKE 1A* (*TDL1A*), and *ERECTA2* (*ER2*) are of great importance in regulating the number of cells involved in female sporogenesis in rice. These three genes ensure that a single sub-epidermal cell in the ovule nucellus forms a single female AC, which then differentiates into an EMC and an FM (Nonomura *et al.*, 2003; Zhao *et al.*, 2008, 2020). MEIOTIC RECOMBINATION 11 (MRE11) regulates DNA replication and damage repair of the mitotic cell cycle before meiosis, ensuring that the EMC develops normally in rice (Shen *et al.*, 2021). *MEIOSIS ARRESTED AT LEPTOTENE1* (*MEL1*), which belongs to the ARGONAUTE (AGO) family, is essential for pre-meiotic mitosis and meiosis. The Tos17 insertion in this gene leads to the interruption of meiosis in the EMC, preventing the development of viable embryo sacs (Nonomura *et al.*, 2007).

Many genes have been reported to be involved in embryo sac development in rice. *HOMOLOGOUS PAIRING ABERRATION IN RICE MEIOSIS1* (*PAIR1*), *PAIR2*, *PAIR3*, and *POOR HOMOLOGOUS SYNAPSIS 1* (*PHS1*) are either specifically or strongly expressed at the EMC stage, and play crucial roles in controlling homologous chromosome pairing during meiosis (Nonomura *et al.*, 2004a, 2004b; Yuan *et al.*, 2009; Yu *et al.*, 2022). Mutations in these genes cause the embryo sac to lack any discernible structure. MEIOTIC RECOMBINATION PROTEIN8 (REC8), a key component of the meiotic cohesion complex in rice, affects meiosis by regulating chromatid cohesion and the monopolar orientation of the kinetochore in meiosis I (Shao *et al.*, 2011). *REPLICATION PROTEIN A* (*RPA1a*), *X-RAY REPAIR CROSS-COMPLEMENTING PROTEIN 3* (*XRCC3*), (*RADIATION SENSITIVE 1*) *RAD1*, *BIVALENT FORMATION 1* (*P31*^*comet*^), *BREAST CANCER 2* (*BRCA2*), *CHROMATIN REMODELING FACTOR 721* (*CHR721*), *MALE AND FEMALE STERILITY 1* (*MFS1*), and *RADIATION SENSITIVE 51C* (*RAD51C*) influence male and female fertility in rice by facilitating the accurate repair of double-strand breaks and promoting homologous recombination during the meiotic process (Chang *et al.*, 2009; Kou *et al.*, 2012; Tang *et al.*, 2014; Zhang *et al.*, 2015, 2020; Hu *et al.*, 2016; Ji *et al.*, 2016; Fu *et al.*, 2020; J.Y. Lu *et al.*, 2020). The leptotene phase of meiotic prophase I is controlled by *TYPE-B RESPONSE REGULATOR 4* (*LEPTO1*), also called as *DEFECTIVE LEPTOTENE CHROMOSOME* (*DLC1*), and mutation in this gene cause male and female sterility (Zhao *et al.*, 2018; Tao *et al.*, 2019). *MUTS-HOMOLOG 5* (*MSH5*), *SHORTAGE IN CHIASMATA1* (*SHOC1*), and *ABERRANT GAMETOGENESIS 1* (*AGG1*) are all necessary for crossover formation during rice meiosis, and mutations in these genes again cause male and female sterility (Luo *et al.*, 2013; Ren *et al.*, 2019; Chang *et al.*, 2020). A TPR repeat-containing protein (ZIP4) is required for homologous chromosome synapsis and crossover formation in rice meiosis, and mutations have been seen to produce sterile gametes in both males and females (Shen *et al.*, 2012). *MTOPVIB*, *PRD2*, and *SDS* are characterized as affecting male and female development through their involvement in double-strand break formation in rice meiosis (Wu *et al.*, 2015; Xue *et al.*, 2016; Wang *et al*., 2023). The DNA mismatch repair genes *MLH3* and *MSH4* are involved in the formation of type I crossovers and the regulation of megaspore development. The rice mutants *mlh3* and *msh4* exhibit abnormalities in the megaspore during the tetrad stage, resulting in the inability to create functional megaspores (Wang *et al.*, 2015; Mao *et al.*, 2021). In the FM stage, a knockout transgenic mutant of *DEFECTIVE EMBRYO SAC1* (*DES1*) has been shown to exhibit an undifferentiated mature embryo sac similar to the mutant *des1*, confirming that the disorder occurring in the formation of FMs in *des1* is attributable to *DES1* mutation (Hu *et al.*, 2023). In addition, *EMBRYO SAC ABORTION 1* (*ESA1*) has been identified as participating in embryo sac degeneration during early mitosis in an interspecific rice hybrid (Hou *et al.*, 2019). When *DEFECT IN EARLY EMBRYO SAC1* (*DEES1*) is suppressed through RNAi in rice, the FM is unable to undergo mitosis, and this leads to degeneration and emptiness of the embryo sac during female gametogenesis (Wang *et al.*, 2012). *EMBRYO SAC DEVELOPMENT 1* (*ESD1*) and *ANAPHASE-PROMOTING COMPLEX 6* (*APC6*) also regulate the process of cell division and differentiation during megagametophyte development, and rice *esd1* mutants exhibit degraded egg cells during embryo sac differentiation, while an *APC6* mutant with a T-DNA insertion shows either a reduced number or lack of polar nuclei, ending up with seven nuclei instead of eight (Awasthi *et al.*, 2012; Wang *et al.*, 2021). In addition, microRNAs (miRNAs) act as important regulators for female reproductive development. Overexpressing miRNA5488, a rice species-specific miRNA, causes embryo sac abortion whilst no effect is observed when miRNA5488 is silenced (Guo *et al.*, 2023). Similarly, overexpression of miRNA5506 in rice plants leads to anomalies in both the embryo sac and floral organs (Chen *et al.*, 2021).

DEAD-box protein is an ATP-independent RNA helicase found in all eukaryotic organisms (Linder *et al.*, 1989; Gorbalenya and Koonin, 1993) and it takes part in various aspects of RNA metabolism, such as pre-mRNA splicing, ribosome biogenesis, RNA translation, and decay (Jarmoskaite and Russell, 2011; Linder and Jankowsky, 2011). In plants, many of the DEAD-box superfamily members are involved in the regulation of abiotic and biotic stress responses, growth, and development, such as *DEAD31* and *DEAD39* in *Solanum lycopersicum* (Zhu *et al.*, 2015; Capel *et al.*, 2020), *DHC1* in *Brassica rapa* (Cao *et al.*, 2022), *DEADRH25a* in *Vitis vinifera* (Yang *et al.*, 2022), and *RH3* and *RH7*/*PRH75* in Arabidopsis (Gu *et al.*, 2014; Huang *et al.*, 2016a). Two genes related to DEAD-box proteins regulate embryo sac development in Arabidopsis have been characterized, namely *RNA HELICASE 36* (*RH36*; also called *SWA3*) and *RH29* (Huang *et al.*, 2010a; Liu *et al.*, 2010; Chen *et al.*, 2020). According to the Rice Annotation Project Database (https://rapdb.dna.affrc.go.jp/), there are 56 genes belonging to the DEAD-box RNA helicase family in rice (Supplementary Fig. S1A). Among them, 12 have been studied, namely *BIRH1* (Li *et al.*, 2008), *TCD33* (*RH50*; X. Wang *et al.*, 2020), *RH17* (Xu *et al.*, 2015), *TOGR1* (*RH10*; Wang *et al.*, 2016), *ABP* (*RH31*; Macovei *et al.*, 2012), *RH53* (Nawaz *et al.*, 2018), *RH58* (Nawaz and Kang, 2019), *RH42* (C.A. Lu *et al.*, 2020), *AIP1* (*RH56*), *RH15* (*AIP2*; Li *et al.*, 2011), *RH2*, *RH34* (Huang *et al.*, 2016b), and *RH36* (Huang *et al.*, 2010b). *BIRH1* (*TCD33*) is involved in responses to pathogen infection and oxidative stress (Li *et al.*, 2008) and in early chloroplast development via the regulation of cold tolerance in rice (X. Wang *et al.*, 2020). Expression of *OsRH17* in *Escherichia coli* inhibits 16S ribosomal RNA maturation (Xu *et al.*, 2015). Nucleolar-localized THERMOTOLERANT GROWTH REQUIRED1 (TOGR1; RH10) has been shown to possess pre-rRNA chaperone activity, improving rice growth at high temperatures (Wang *et al.*, 2016). *ATP-BINDING PROTEIN* (*ABP*) plays an important role in abiotic stress responses (Macovei *et al.*, 2012). When expressed in Arabidopsis, *OsRH53* has a negative impact on responses to abiotic stresses including drought, salt, cold, and UV stress, and the transgenic plants show delayed seed germination and reduced growth under high salinity or dehydration stress (Nawaz *et al.*, 2018). In contrast, transgenic expression of *OsRH58* promotes seed germination, seedling growth, and survival rates in Arabidopsis under salt or drought stress (Nawaz and Kang, 2019). *RH42* in rice is essential for pre-RNA splicing and improves plant growth under cold temperatures (C.A. Lu *et al.*, 2020). DEAD-box proteins also participate in reproductive development in rice. *APOPTOSIS INHIBITOR1* (*AIP1*) and *AIP2* mediate the expression of *AIP5*, leading to the postponement of tapetum degeneration via programmed cell death (Li *et al.*, 2011). Double-knockdown of *RH2* and *RH34*, two highly similar DEAD-box proteins results in dwarfism, aborted pollen grains, and low seed set (Huang *et al.*, 2016b). Our current understanding of the involvement of DEAD-box proteins in the regulation of gametogenesis in rice is limited. The rice mutant *rh36* exhibits a phenotype similar to its Arabidopsis counterpart, indicating that it has a potential role in embryo sac development (Huang *et al.*, 2010b); however, the precise biological functions of rice DEAD-box proteins in this context remain unclear.

In previous research, we found that a DEAD-box RNA helicase family member, *RH52A*, affects seed setting in neo-tetraploid rice, and the coding sequence region of the neo-tetraploid was 27 bp less than that of diploid rice (Yu *et al.*, 2020; Supplementary Fig. S1B); however, the function of *RH52A* in controlling fertility remained unclear in diploid rice. In the present study, knockout mutants of *RH52A* were developed in the diploid *japonica* cultivar Nipponbare using CRISPR/Cas9 gene-editing technology, and they displayed normal pollen fertility but low seed setting. Detailed examination indicated that the primary factor contributing to the decrease in seed setting in the *rh52a* mutants was reduced fertility of the embryo sac. Cytological observations revealed that double-female-gametophytes developed from the two chalazal FMs, and ~3% of these were fertilized through the sac close to the micropylar end. Several differentially expressed genes associated with embryo sac development were identified in the mutant by RNA-sequencing analysis. The results of our study provide substantial evidence that the DEAD-box RNA helicase family is crucial in regulating female reproductive development in rice.

## Materials and methods

### Plant materials and growth conditions

Experiments were performed using the wild type *Oryza sativa* subsp. *japonica* cultivar Nipponbare and two mutants (*rh52a-m1* and *rh52a-m2*) that were created using the CRISPR/Cas9 gene-editing method.

The targetDesign tool of CRISPR-GE (http://skl.scau.edu.cn/home/) was utilized to design two pairs of guide RNAs for exons 1 and 3 of the *OsRH52A* gene (Xie *et al.*, 2017). The knockout vector was constructed as described previously (Ma *et al.*, 2015). The vector was transformed into Nipponbare by *Agrobacterium*-mediated transformation, and the mutated plants (*rh52a*) were detected by Sanger sequencing using gene-specific target primers. Primers for vector construction are listed in Supplementary Table S1. F_1_ hybrids were developed by crossing the mutant (*rh52a-m2*) with wild type Nipponbare, and F_2_ generations were subsequently acquired by performing self-crossing of the F_1_ hybrids.

Plants were grown in an experimental field at the South China Agricultural University in Guangzhou according to standard agricultural practices.

### Determination of pollen fertility, viability, and germination *in vivo*

When the anther reached to the top of the spikelet, pollen fertility was determined by staining the grains with 1% iodine/potassium iodide (I_2_–KI) solution. Pollen grains densely stained were counted as fertile, while those unstained or shrunken were regarded as sterile (Ghouri *et al.*, 2019). Alexander’s staining solution (Harveybio Gene Technology Co. Ltd, Beijing, China) was used to test the pollen viability, with purple staining indicating they were viable, and blue that they were unviable (Huang *et al.*, 2010a). In both cases, the pollen grains were observed under a Motic BA210 light microscope. At least 500 grains were observed for each sample.

Self-pollinated spikelets were sampled at 5 min after pollination, and fixed for 24 h in FAA (50% absolute ethanol/formalin/acetic acid, 89:5:6). Pollen germination *in vivo* was determined following the protocol of Shi *et al.* (2018). The pollinated pistils were dissected, processed through an ethanol series (70, 50, and 30%; 20 min each), and washed three times with distilled water. They were then incubated in 1 mol l ^–1^ sodium hydroxide for 1 h at room temperature, washed three times with distilled water, and stained in 0.1% Aniline blue solution for 12 h in the dark. The pollinated pistils were imaged using a fluorescence microscope (Leica DMRXA).

### Embryo sac fertility and development

Spikelets were sampled at the following stages: AC, EMC prior to meiosis, EMC at meiosis, the mitosis stage of the embryo sac, the mature stage (flowering), 1 day after fertilization (DAF), and 3 DAF. The spikelets were immersed in FAA solution for 24 h. After being washed three times with 50% ethanol, development was assessed using whole-mount eosin B-staining/confocal laser-scanning microscopy (WE-CLSM), as described previously (Zeng *et al.*, 2007; Ghouri *et al.*, 2019). In brief, dissected ovaries were rehydrated with an ethanol series (50%, 30%, and 10%; 20 min each), washed with distilled water three times, and treated for 20 min with 2% aluminum potassium sulfate dodecahydrate as a mordant. The ovaries were then stained in 1% eosin B for 12 h, treated again with 2% aluminum potassium sulfate dodecahydrate for 20 min, and dehydrated with a graded ethanol series (0, 10, 30, 50, 70, 90, and 100%; 20 min each). The ovaries were incubated overnight in a mixture of absolute ethanol and methyl salicylate (1:1) and transferred to 100% methyl salicylate before observation under a Leica TCS SPE confocal laser-scanning microscope.

Semi-sectioning was performed to characterize the embryo sac development of the ovaries in the wild type and mutants as described by Z.Y. Lu *et al.*, (2020). The samples were embedded in Technovit^®^ 7100 resin (Hereaus Kulzer, Wehrheim, Germany), polymerized at 65 °C, and cut into 3 μm sections using a rotary microtome. The sections were stained with Toluidine blue O and imaged using the Motic BA210 light microscope.

### Histochemical analysis of GUS activity

A construct *pCAMBIA1305::GUS*, in which the *β-glucuronidase* (*GUS*) reporter gene was driven by the native *OsRH52A* promoter, was introduced into Nipponbare by *Agrobacterium*-mediated transformation.

Spikelets were incubated at 37 °C in a GUS staining solution (Leagene Biotechnology, Beijing, China). The stained tissues were transferred to a 70% (v/v) ethanol solution for clearing, and then imaged using a Leica MZ16 stereoscope.

### RNA extraction and qRT-PCR

Total RNA was extracted from fresh samples using AG RNAex Pro Reagent (Accurate Biotechnology, Hunan, China) following the manufacturer’s instructions. Then, 1 μg of total RNA of each sample was reverse-transcribed to cDNA using an Evo M-MLV RT Kit with gDNA Clean for qPCR (Accurate Biotechnology). Real-time quantitative (qRT-)PCR was performed using a Hieff^®^ qPCR SYBR Green Master Mix Kit (Yeasen Biotechnology, Shanghai, China) on a Roche LightCycler^®^ 480 Instrument II (96-multiwell plates). The rice *UBIQUITIN* gene (*Os03g0234200*) and *CYTOSOLIC TRIOSEPHOSPHATE ISOMERASE* (*CPI*; *Os01g0147900*) were used as reference genes. *CPI* is a stable reference gene, as reported by Wu *et al.* (2021). Each sample consisted of RNA extracted from at least three individual plants pooled together, and three biological replicate samples were analysed. Relative expression was determined using the 2^(−ΔΔ*C*T)^ method (Livak and Schmittgen, 2001; Mao *et al.*, 2021). The primers used for qRT-PCR are listed in Supplementary Table S1.

### Subcellular localization assays in tobacco

The coding sequence of *OsRH52A*, excluding the stop codon, was successfully inserted into two different vectors, namely pBin19-EGFP and pOX-GFP. Primers for vector construction are listed in Supplementary Table S1. The *pBin19::OsRH52A::EGFP* fusion construct was introduced into the *Agrobacterium* strain GV3101 for transient expression in leaves of tobacco (*Nicotiana benthamiana*). The GFP fluorescence signal was observed 3–4 d after transformation. The vector containing the *pOX::OsRH52A::GFP* construct was transformed into rice protoplasts using the polyethylene glycol (PEG)-mediated transformation method (Chen *et al.*, 2006). After 12–16 h cultivation, the GFP fluorescence signals were detected at an excitation wavelength of 488 nm using a Zeiss LSM 7180. The pCAMBIA2300-mCherry and PAN580-mKATE constructs were used as nuclear markers in the tobacco leaves and rice protoplasts, respectively. All primers are shown in Supplementary Table S1.

### Yeast two-hybrid assays

The full-length coding sequence of *OsRH52A* was cloned into the pGBKT7 vector and those of *OsMFS1* and *OsZIP4* were each cloned into pGADT7. pGBKT7-OsRH52A was transferred into the yeast strain Y2H Gold and grown on SD/–Trp and SD/–Trp–His media for toxicity and autoactivation tests. BD-OsRH52A served as the bait vector and AD-OsMFS1 and AD-ZIP4 as prey vectors, and they were co-transformed into Y2H Gold and grown on SD/–Leu–Trp medium at 30 °C for 2 d, and then transferred to SD/–Trp–Leu–His–Ade medium and cultivated at 30 °C for 2–4 d. The primers for the yeast two-hybrid assays are listed in Supplementary Table S1.

### Bimolecular fluorescence complementation assays

For bimolecular fluorescence complementation (BiFC) assays, the coding sequence of *OsRH52A* was cloned into the p2YN vector, yielding the *OsRH52A::p2YN* construct, while those of *OsMFS1* and *OsZIP4* were each cloned into the p2YC vector to generate *OsMFS1::p2YC* and *OsZIP4::p2YC*. The primers are listed in Supplementary Table S1. Empty vectors and fusion proteins were transiently expressed by Agro-infiltration in tobacco leaf epidermal cells (He *et al.*, 2023), and fluorescence of yellow fluorescent protein (YFP) was observed using the Leica TCS SPE confocal laser-scanning microscope.

### Firefly luciferase complementation imaging assays

Luciferase (LUC) complementation imaging assays for interactions between OsRH52A and OsMFS1, as well as OsZIP4 were performed in tobacco leaves. The full-length *OsRH52A* was fused with the C-terminus of *LUC*, whilst the coding regions of *OsMFS1* and *OsZIP4* were each fused N-terminus of *LUC*. The recombinant plasmids cLUC-OsRH52A, OsMFS-nLUC, ZIP4-nLUC, and empty vectors were transformed into *Agrobacterium* strain GV3101. After culturing, cells of GV3101 were collected and resuspended in infiltration buffer (10 mM MgCl_2_, 100 mM acetosyringone, 10 mM MES-KOH, pH 5.6) to a final OD_600_ of 0.8 and then co-injected into tobacco leaves (J. Wang *et al.*, 2020). The leaves were imaged after 48 h to detect the LUC signals using a NightSHADE evo LB 985 In Vivo Plant Imaging System (Berthold). All primers used for vector construction are presented in Supplementary Table S1.

### RNA-sequencing experiments and data analysis

RNA isolated from ovaries at the meiotic and FM stages of the wild type (WT) and the knockout mutant *rh52a-m2* was used for RNA-seq. Three biological replicates were conducted for the RNA extraction, each of which consisted of 25 mg of tissue collected from at least three plants. For transcriptome sequencing analysis, all clean reads were aligned to the reference genome of *O. sativa* subsp. *japonica* cv. Nipponbare (RGAP 7; http://rice.uga.edu/) using software HISAT 2.0. Differences in gene expression between WT versus *rh52a-m2* were detected using the DESeq2_edgeR package. Differentially expressed genes (DEGs) were determined based on the threshold of a false-discovery rate adjusted *P*-value ≤0.05 and an absolute value of log_2_|fold-change| ≥1.0. The identified DEGs were used for analyses of GO enrichment (http://systemsbiology.cau.edu.cn/agriGOv2/index.php) and KEGG enrichment (https://www.kegg.jp/). Analysis of the degradation pathway of DEGs was performed using the MapMan software. Prediction of protein–protein interactions was performed using the STRING database (http://string-db.org/).

## Results

### Rice *RH52A* is a member of DEAD-box RNA helicase and is highly expressed in the ovary

**Fig. 1.**
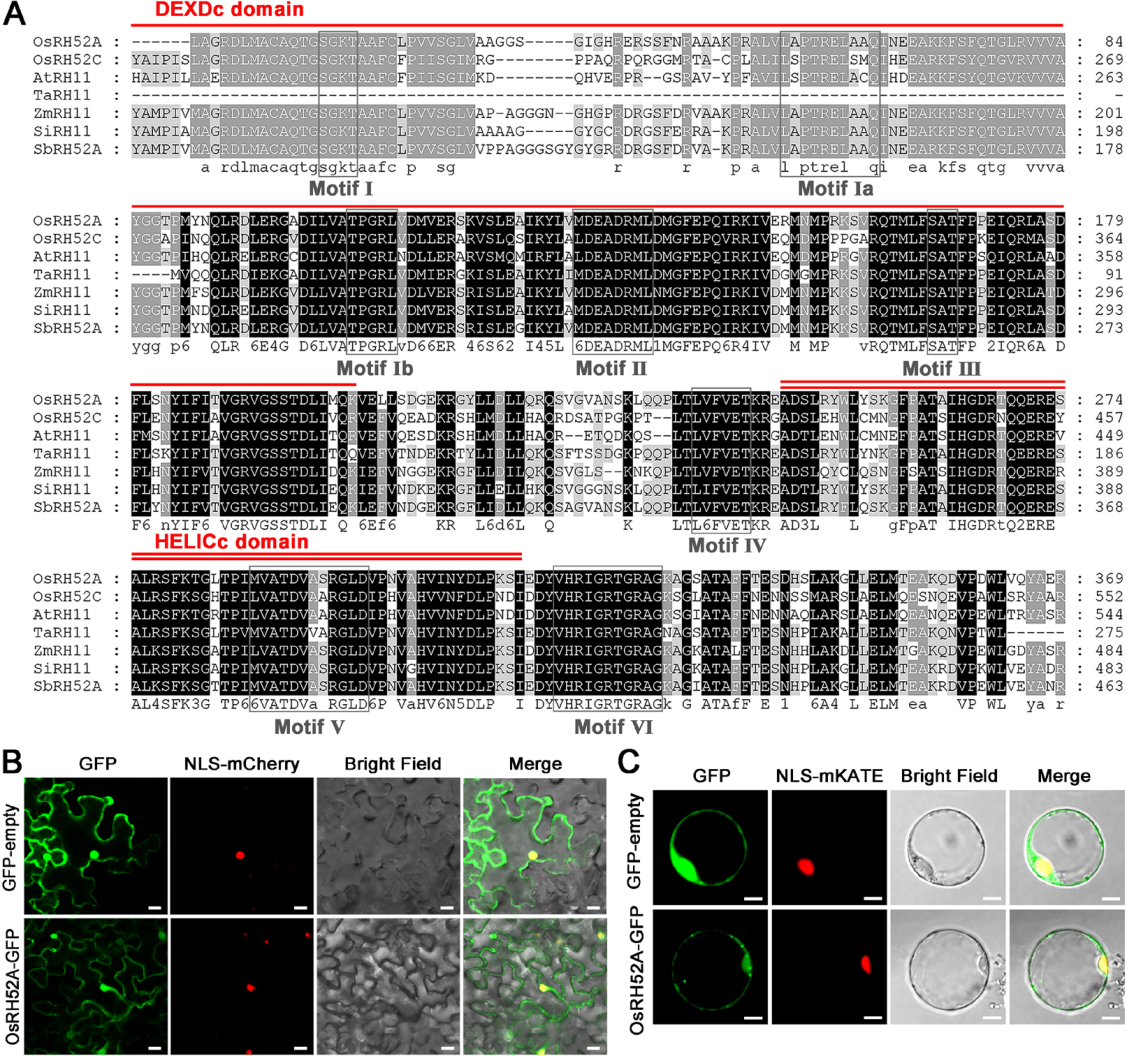
Homology analysis and subcellular localization of OsRH52A. (A) Multiple amino acid sequence alignment of proteins from the DEAD-box RNA helicase family in *Oryza sativa* (OsRH52A, OsRH52C), *Arabidopsis thaliana* (AtRH11), *Triticum aestivum* (TaRH11), *Zea mays* (ZmRH11), *Setaria italic* (SiRH11), and *Sorghum bicolor* (SbRH52A). The red lines indicate the DEXDc and HELICc domains of OsRH52A, whilst the eight motifs of DEAD-box RNA helicase are indicated by the black rectangles. (B) The subcellular localization of the OsRH52A protein in rice protoplasts. GFP, green fluorescence protein. ‘Merge’ is the overlapped image of GFP, the nuclear marker NLS-mCherry, and the bright field. Scale bars are 20 μm. (C) The subcellular localization of the OsRH52A protein in leaves of *Nicotiana benthamiana*. NLS-mKATE was used as the nuclear marker. Scale bars are 40 μm.

**Fig. 2.**
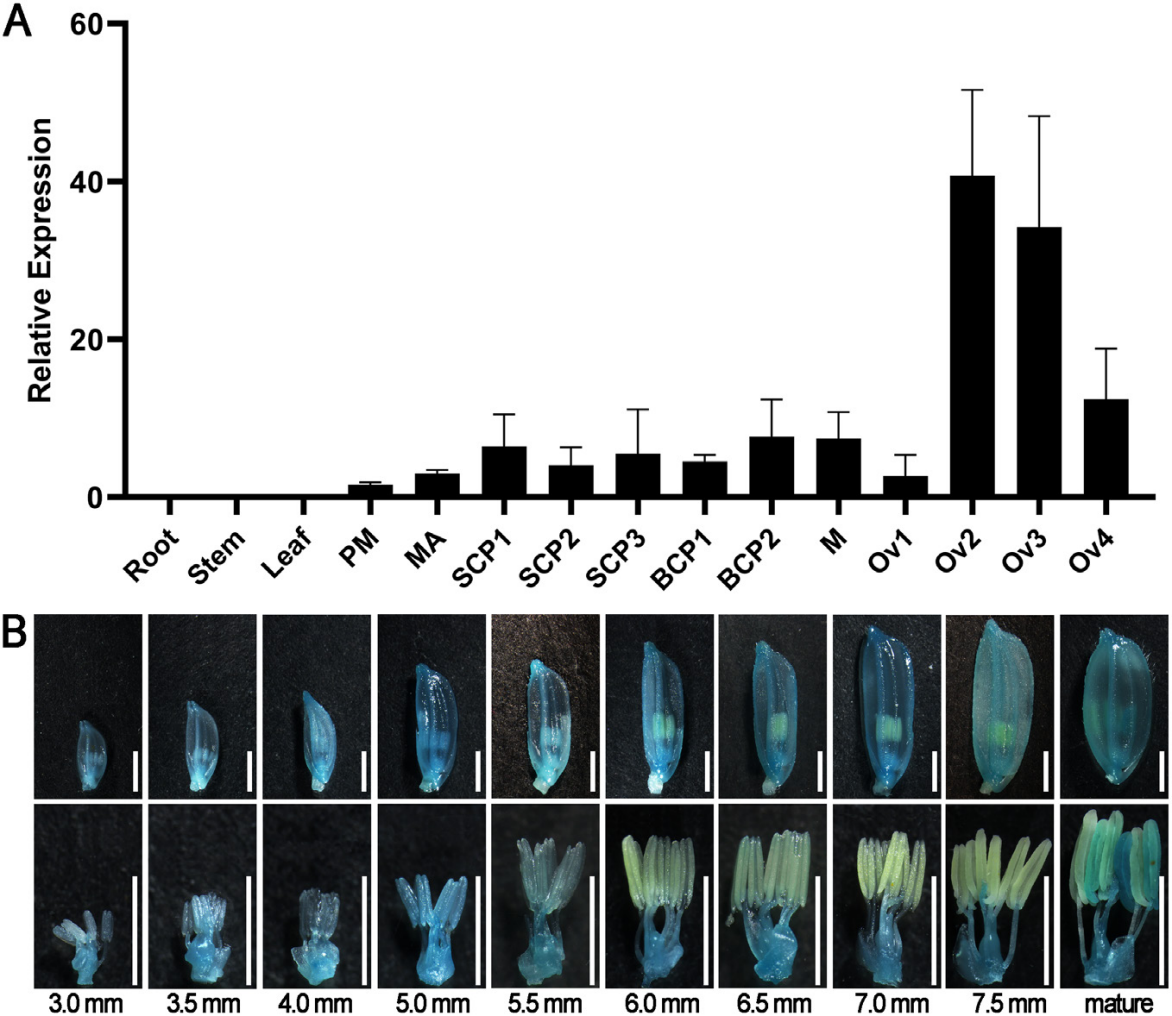
Expression patterns of *RH52A* in different tissues of wild type Nipponbare rice. (A) Relative expression of *RH52A* in the roots, stem, leaves, and developing spikelets. PM, anther and ovary at the pre-meiosis stage; MA, anther at meiosis stage; SCP1, early microspore stage; SCP2, middle microspore stage; SCP3, late microspore stage; BCP1, early bicellular pollen stage; BCP2, late bicellular pollen stage; M, mature anther. Ov1, ovary at meiosis of embryo sac (ES); Ov2, ovary at functional megaspore stage of ES; Ov3, ovary at mitosis of ES; Ov4, ovary of mature ES. (B) Histochemical staining of developing spikelets (top) and their corresponding reproductive organs (bottom) expressing the *GUS* reporter gene driven by the *RH52A* promoter. The length of the developing spikelets is indicated below the images. Scale bars are 2 mm.

Sequence analysis revealed that rice *RH52A* (*LOC_Os06g40020*) has a length of 1437 bp and comprises six exons (Supplementary Fig. S1B). This genetic sequence encoded 479 amino acids in the Nipponbare variety (http://rice.uga.edu/cgi-bin/ORF_infopage.cgi?orf=LOC_Os06g40020). A phylogenetic tree constructed with all 56 DEAD-box RNA helicase proteins in rice indicated that RH52A belongs to the DEAD-box RNA helicase family (Supplementary Fig. S1A). Protein sequence alignment demonstrated that *RH52A* contains a DEXDc and a HELICc domain with a total of eight conserved RNA helicase motifs (Fig. 1A). In addition, analysis of a phylogenetic tree constructed of proteins in *Gramineae* revealed that OsRH52A shared the closest similarity with *Oryza brachyantha* (XP_04038129.1) (Supplementary Fig. S2). Examination of subcellular localization showed that OsRH52A was present in the nucleus and cytoplasm of tobacco epidermal cells (Fig. 1B), and this was consistent with results obtained in rice protoplasts (Fig. 1C). qRT-PCR analysis indicated that the expression of *RH52A* in rice was relatively low in the roots, stem, and leaf tissues, at moderate levels in the anthers, but much higher in the ovaries, particularly at the functional megaspore and mitosis stages (Fig. 2A). These findings were compatible with the observations obtained from GUS staining, where maximum GUS activity was detected when spikelets were 6–7.5 mm in length (Fig. 2B). These results suggested that *RH52A* has a potential function in the embryo sac development of rice.

### Knockout of *RH52A* results in low seed set

**Fig. 3.**
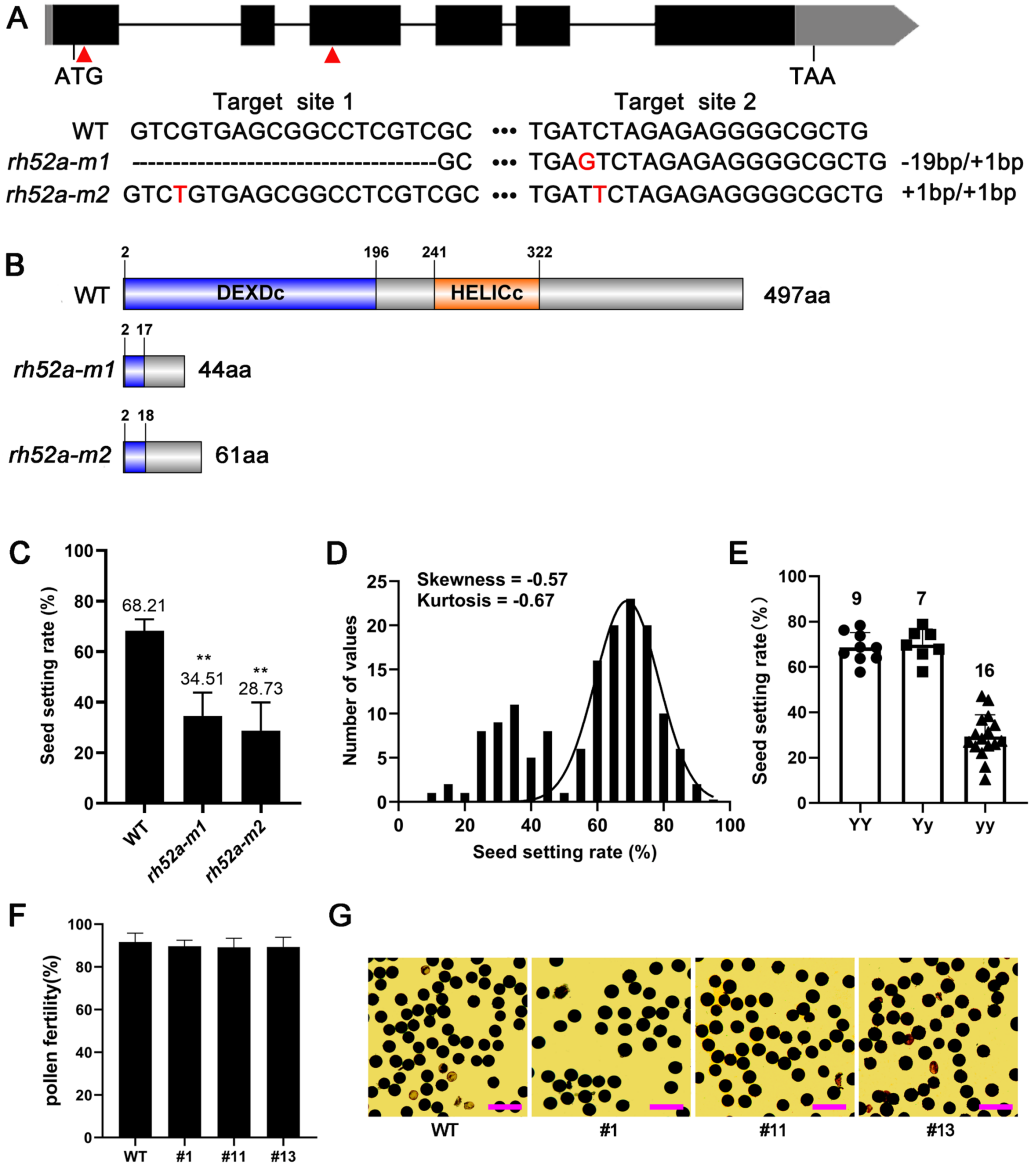
CRISPR/Cas9 mutation of rice *RH52A* and analysis of its effects on seed setting and pollen fertility. (A) Nucleotide alignments of the CRISPR/Cas9 target sites in the Nipponbare wild type (WT) and the two mutants *rh52a-m1* and *rh52a-m2*. In the schematic diagram, the lines represent the introns and the black boxes show the exons; grey boxes indicate the untranslated regions. The two target sites are indicated. (B) Amino acid alignments of RH52A in the WT and the *rh52a-m1* and *rh52a-m2* mutants. (C) Seed setting rate of the WT and the *rh52a-m1* and *rh52a-m2* mutants. Values are means (±SD), *n*=15. Significant differences compared with the WT were determined using Student’s *t*-test: **P<0.01. (D) Frequency distribution of F_2_ populations from the cross between *rh52a-m2* and the WT. (E) Seed setting rates of 32 randomly selected lines from the F_2_ population that were genotyped by Sanger sequencing. (F) Pollen fertility of the WT and three randomly selected homozygous lines (yy) from the F_2_ population. Values are means (±SD), *n*=3. (G) Pollen fertility of the WT and the three homozygous lines (yy) using I_2_-KI staining assays. Scale bars are 100 μm.

In our previous study using transcriptome analysis conducted on neo-tetraploid rice, a total of 81 genes were identified as exhibiting elevated expression levels in reproductive organs (Yu *et al.*, 2020). Here, we compared these genes to 5366 that have previously been reported for expression in reproductive tissues in rice (Supplementary Table S2), and found that a DEAD-box protein, RH52A, interacted with 13 proteins related to fertility (Supplementary Fig. S3), suggesting that it might regulate fertility in diploid rice. To examine this, we used CRISPR/Cas9 gene-editing technology to knock out *RH52A*, and two stable homozygous mutants (*rh52a-m1* and *rh52a1-m2*) were obtained in the T_3_ generation. The mutant *rh52a-m1* contained a deletion of the sequence ‘CGTCGTGAGCGGCCTCGTC’ at target site 1 (TS1) and an insertion of ‘G’ at target site 2 (TS2), whilst *rh52a-m2* contained an insertion of ‘T’ at both TS1 and TS2 (Fig. 3A). These InDel mutations changed the translated protein sequence after 17 amino acids (aa) in *rh52a-m1* and 18 aa in *rh52a-m2*, and early terminations occurred after 44 aa in *rh52a-m1* and 61 aa in *rh52a-m2* (Fig. 3B). These amino acid sequence changes led to the loss of the DEXDc and HELICc domains. In the T_3_ generation *rh52a-m1* and *rh52a-m2* displayed significant reductions in seed set, with rates of 34.51% and 28.73%, respectively (Fig. 3C).

All F_1_ plants derived from a cross between the *rh52a-m2* mutant and Nipponbare displayed a WT phenotype. The F_2_ progeny presented a skew–normal distribution, which was distinguished by values <1 for skewness and kurtosis (absolute value) for seed set (Fig. 3D). This demonstrated that *RH52A* is a prominent gene responsible for determining seed set. A total of 32 individual plants from the F_2_ population were selected randomly for genotype detection using Sanger sequencing. Homozygous dominant and heterozygous plants exhibited the same seed-setting rate as the WT, while homozygous recessive plants were consistent with the *rh52a* mutants (Fig. 3E). Three representative homozygous individual plants were then selected for examination, and their mature pollen grains showed no significant differences in their pollen fertility compared with the WT (Fig. 3F, G). These results demonstrated that the functional loss of *RH52A* caused low fertility that was not due to reduced pollen fertility.

### Mutation of *RH52A* causes abnormal embryo sac development and low fertility

**Fig. 4.**
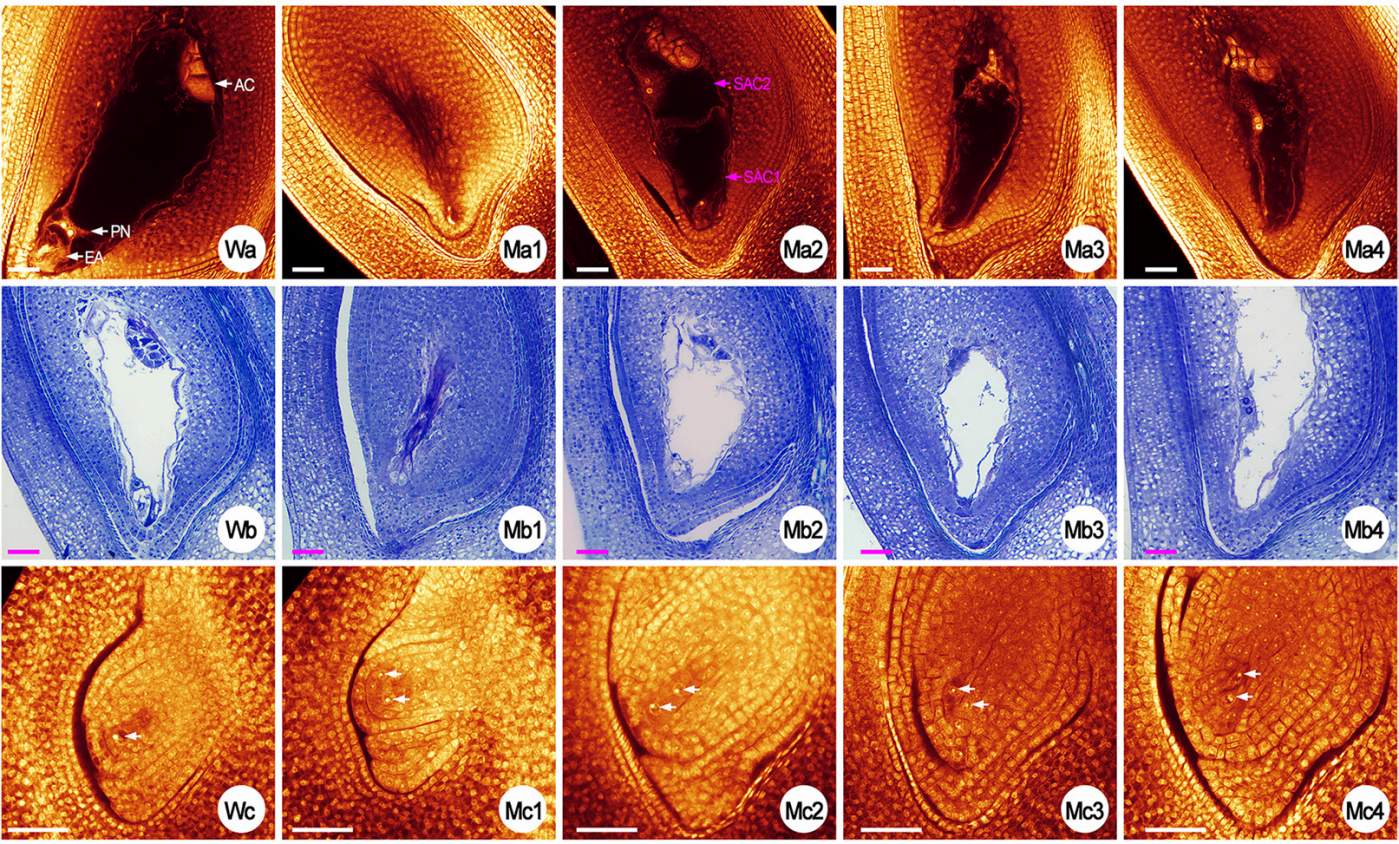
Representative images of various abnormal embryo sacs of the rice wild type (WT) and the *rh52a-m2* mutant. Red embryo sacs were obtained by whole-mount eosin B-staining/confocal laser-scanning microscopy (WE-CLSM). Blue embryo sacs were obtained from semi-thin sections stained with Toluidine blue O. Wa and Wb show normal mature embryo sacs of the WT. Ma1 and Mb1 show embryo sac degeneration of the mutant. EA, egg apparatus (consisting of an egg cell and two synergids); PN, polar nuclei; AC, antipodal cells. Ma2 and Mb2 show stacked embryo sacs of the mutants. Ma3 and Mb3 show embryo sacs of the mutants without female germ units. Ma4 and Mb4 show embryo sacs of the mutants without egg apparatus. Wc shows the megasporocyte of the WT. Mc1 to Mc4 show multiple megasporocytes of the mutants. The arrows in Wc and Mc1–4 indicate the nuclei. All scale bars are 40 μm.

We next examined pollen and embryo sac fertility in the WT and the *rh52a-m1* and *rh52a-m2* mutants. In all the genotypes, the floral organs were characterized by one inner glume, one outer glume, six stamens, and one pistil (Supplementary Fig. S4). There were more empty spikelets in the *rh52a-m1* and *rh52a-m2* mutants than in the WT (Supplementary Fig. S5A). Three different assays, namely I_2_–KI staining, Alexander staining, and determination of pollen germination *in vivo*, all demonstrated that there were no discernible changes in the pollen fertility, viability, and germination between the two mutants and the WT (Supplementary Figs S5B, S6). However, WE-CLSM observations showed that the fertility of the embryo sac of the two mutants was much lower than the WT, with only 57.3% and 56% normal mature embryo sacs found in *rh52a-m1* and *rh52a-m2*, respectively (Table 1). Four main types of abnormal embryo sacs were found in the two mutants, namely degenerated embryo sacs (21.0% mean value of the two mutants), double-female-gametophytes (two embryo sacs stacked in one ovule together; 11.8%), and embryo sacs without a female germ unit (5.6%) and egg apparatus (3.8%) (Table 1; Fig. 4Ma1–Ma4, Wb1–Wb4). The substantial number of abnormal embryo sacs prevented normal fertilization and resulted in low seed setting, demonstrating that *RH52A* is required for embryo sac development.

**Fig. 5.**
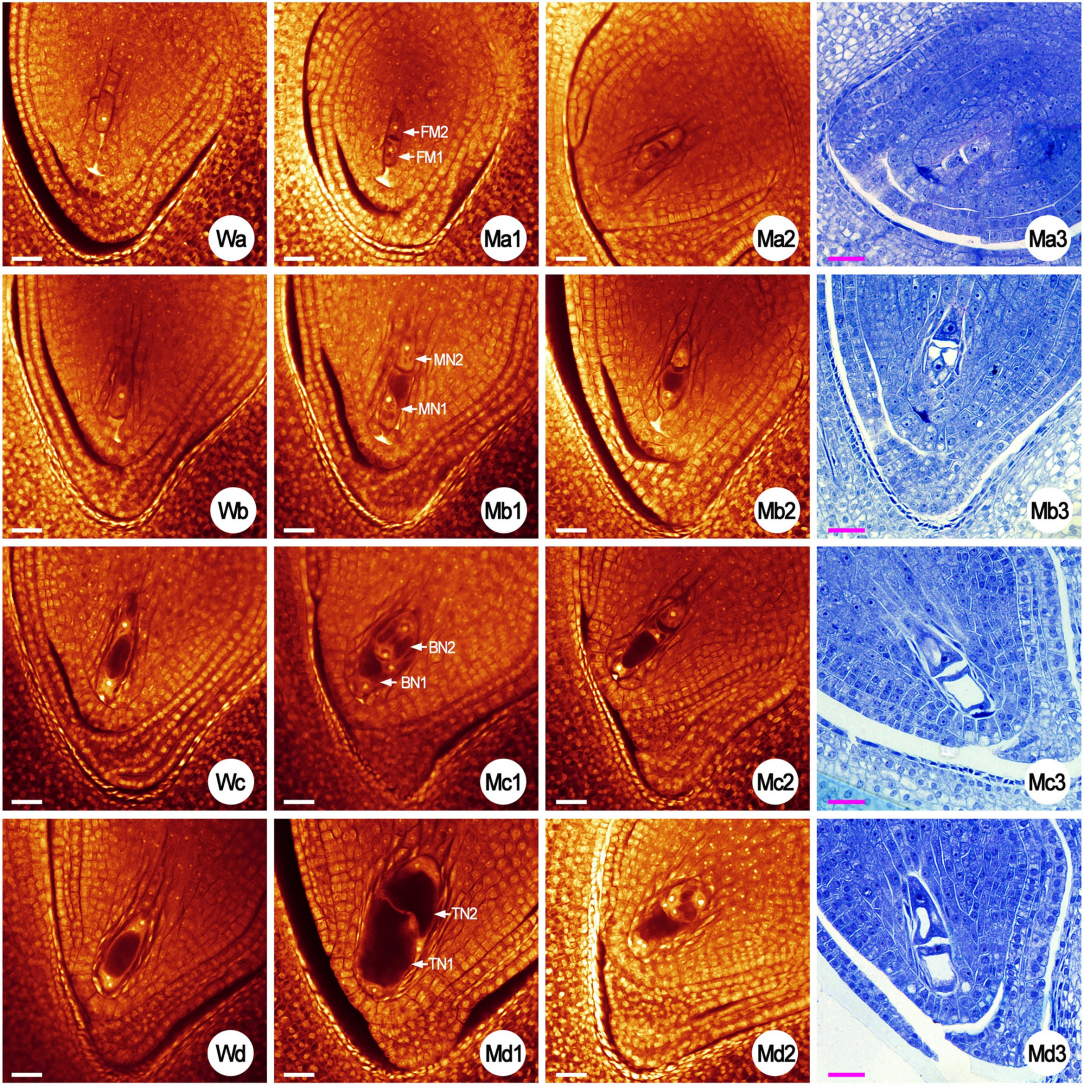
Embryo sac developmental process from the functional megaspore (FM) stage to the tetra-nucleate in the rice wild type (WT) and the *rh52a-m2* mutant. Red embryo sacs were obtained by whole-mount eosin B-staining/confocal laser-scanning microscopy (WE-CLSM). Blue embryo sacs were obtained from semi-thin sections stained with Toluidine blue O. Wa shows the FM of the WT. Ma1 to Ma3 show the double FMs of the mutants. Wb shows the mono-nucleate embryo sac of the WT. Mb1–Mb3 show the double mono-nucleate embryo sacs (MN) of the mutants. Wc shows the bi-nucleate embryo sacs of the WT. Mc1–Mc3, and Md3 show the double bi-nucleate embryo sacs (BN) of the mutants. Wd shows the tetra-nucleate embryo sacs of the WT. Md1 and Md2 show the double tetra-nucleate embryo sacs (TN) of the mutants. All scale bars are 40 μm.

**Fig. 6.**
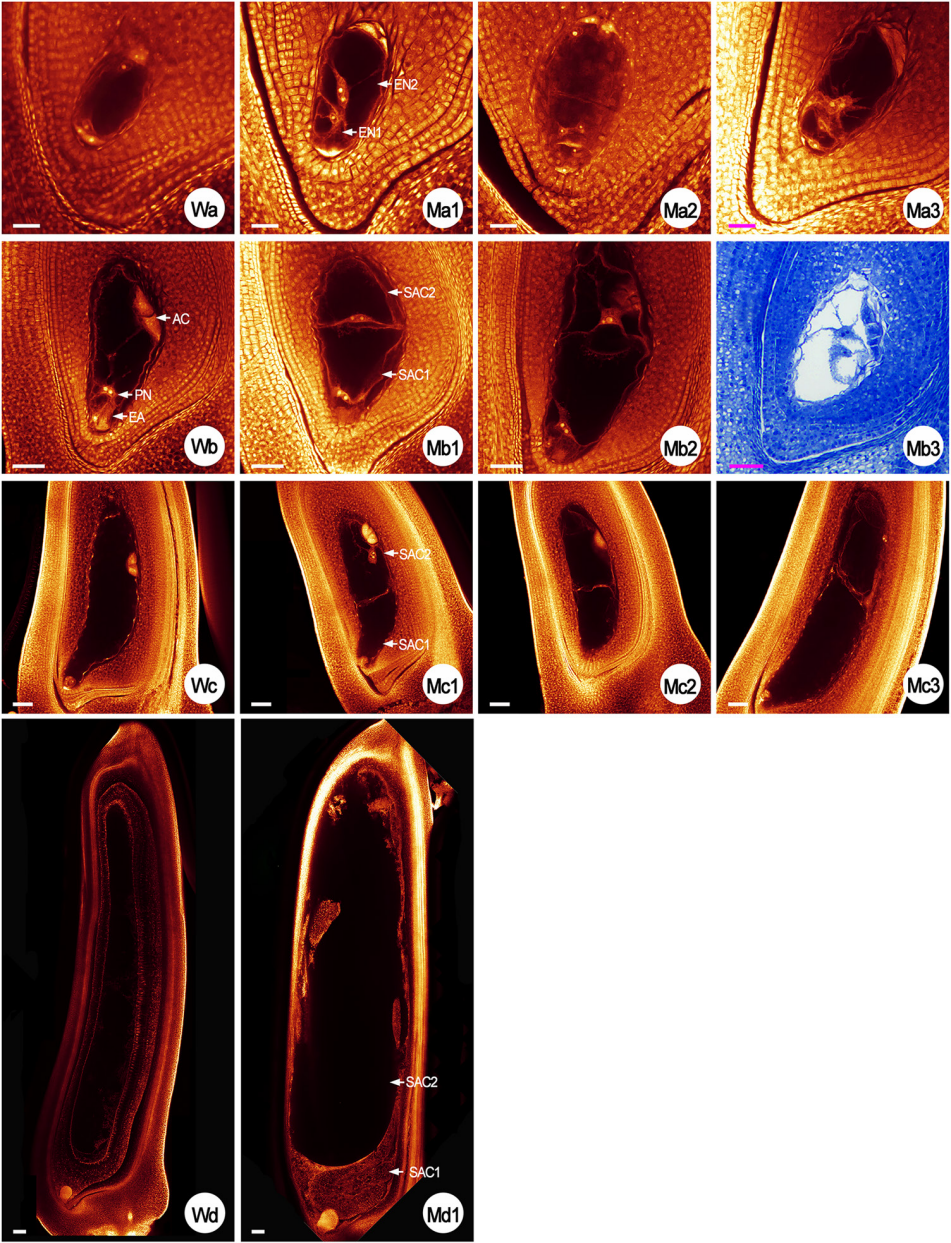
Embryo sac developmental process from the eight-nucleate stage to 3 days after fertilization (DAF) in the rice wild type (WT) and the *rh52a-m2* mutants. Red embryo sacs were obtained by whole-mount eosin B-staining/confocal laser-scanning microscopy (WE-CLSM). Blue embryo sacs were obtained from semi-thin sections stained with Toluidine blue O staining. Wa shows the eight-nucleate embryo sac of the WT. Ma1–Ma3 show the double eight-nucleate embryo sacs (EN) of the mutants. Wb shows the mature embryo sac of the WT. EA, egg apparatus (consisting of an egg cell and two synergids); PN, polar nuclei; AC, antipodal cells. Mb1–Mb3 show the double embryo sacs (SAC) of the mutants. Wc shows the embryo sac of the WT at 1 DAF. Mc1–Mc3 show double embryo sacs of the mutants at 1 DAF. Wd shows the embryo sac of the WT at 3 DAF. Md1 shows the double embryo sacs of the mutant at 3 DAF. Wd and Md1 are compound images constructed from individual segments. Scale bars: Wa, Ma1–Ma3, Wb, Mb1–Mb3=40 μm; Wc, Mc1–Mc3=50 μm; Wd, Md1=100 μm.

**Fig. 7.**
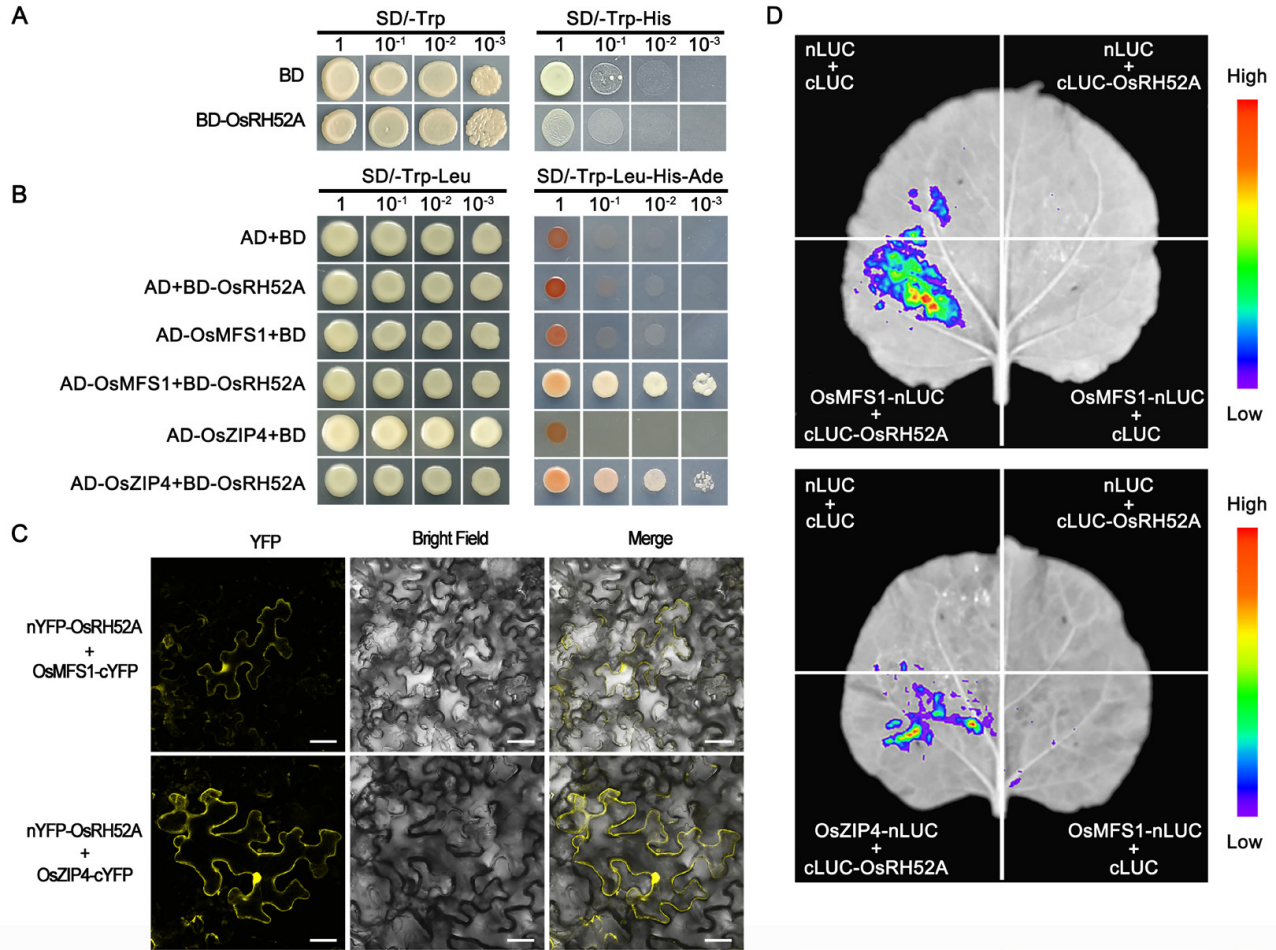
OsRH52A physically interacts with OsMFS1 and OsZIP4. (A) Yeast two-hybrid (Y2H) assays showing no toxicity and autoactivation of the OsRH52A protein. (B) Y2H assays showing the interaction of OsRH52A with OsMFS1 and with OsZIP4. (C) Bimolecular fluorescence complementation assays of OsRH52A with OsMFS1 and OsZIP4 in leaf epidermal cells of *Nicotiana benthamiana*. Scale bars are 40 μm. (D) Luciferase complementation imaging assays showing the interaction of OsRH52A with OsMFS1 and with OsZIP4 in *N. benthamiana* leaves.

**Fig. 8.**
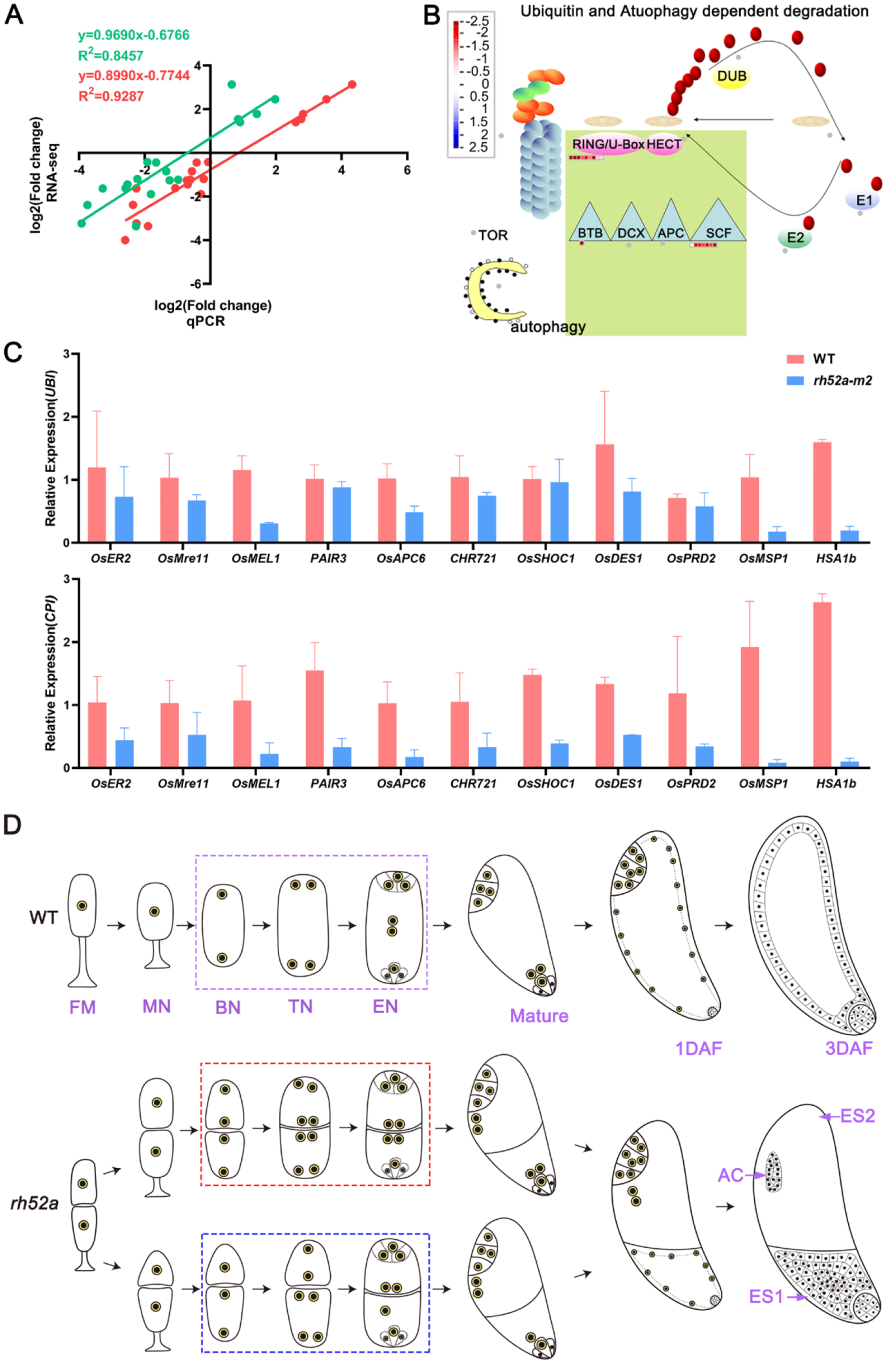
RNA-sequencing analysis and a proposed scheme for the development of double-female-gametophytes in the rice wild type and *rh52a* mutant. (A) Correlation between qRT-PCR analysis and the RNA-seq results for 19 selected genes. The qRT-PCR analysis was carried out using either *UBI* as the reference gene (red circles) or *CPI* (green circles). (B) Mapping of 17 down-regulated differentially expressed genes to ubiquitin degradation pathways in the *rh52a-m2* mutant using the MapMan tool. The relative expression of each of the 17 genes is indicated according to the heatmap scale. (C) The relative expression of genes related to megagametophytes in the wild type (WT) and the mutant at the functional megaspore stage, as determined by qRT-PCR Using either *UBI* or *CPI* as the reference gene. (D) Schematic diagram of the double-female-gametophyte (DFG) developmental process from the functional megaspore to the mature stage, and at 1 day after fertilization (DAF) and 3 DAF. FM, functional megaspore; MN, mono-nucleate embryo sac (ES); BN, bi-nucleate ES; TN, tetra-nucleate ES; EN, eight-nucleate ES. AC, antipodal cells. The embryo sac at the mitosis stage in the wild type (WT) is indicated by the dash box. For the *rh52a* mutant, the dashed red box indicates the process of synchronous division in each sac from DFG at the mitosis stage, whilst the blue box indicates the process of asynchronous division.

**Table 1.** Frequencies of different types of embryo sac observed in rice wild type plants and the two *rh52a* mutants

Genotype	No. ovaries examined	Type of embryo sac (%)				
		NES	ESD	DFG	ESWF	ESWE
Wild type	216	96.3	2.8	0.5	0.5	0.0
*rh52a-m1*	192	57.3	19.8	15.1	3.1	3.1
*rh52a-m2*	248	56.0	22.2	8.5	8.1	4.4
*rh52a* mean	–	56.65	21.0	11.8	5.6	3.8

NES, normal embryo sac; ESD, embryo sac degeneration; DFG, double-female-gametophyte (two embryo sacs stacked in one ovule); ESWF, embryo sac without female germ unit; ESWE, embryo sac without egg apparatus.

The developmental stages of the embryo sac in the two mutants were similar to the WT, including the archesporial cell (AC), megaspore mother cell (MMC, or megasporocyte), dyad, tetrad, functional megaspore (FM), mono-, bi-, tetra-, and eight-nucleate embryo sac (Supplementary Fig. S7), and the mature embryo sac that consisted of an egg cell, two synergids, two polar nuclei, and three antipodal cells (Fig. 4Wa), as has previously been reported (Liu *et al.*, 1997). However, many aberrant megasporocytes and developing gametophytes were detected in the two mutants when compared to the WT, as follows.

(i) Multiple megasporocytes (Fig. 4Mc1–Mc4). Generally, only one MMC developed from the AC and subsequently developed into an embryo-sac mother cell (EMC) (Fig. 4Wc) in the WT, whereas ~2–4 MMCs were observed in each ovary of *rh52a*, with a mean percentage of 5.8% of the embryo sacs examined across the two mutants (Table 2).
(ii) Double FMs (DFMs; Fig. 5Ma1–Ma3). Only one megaspore located towards the chalaza became a FM from the tetrad in the WT (Fig. 5Wa) whereas two cells close to the chalazal end survived at the FM stage and eventually formed DFMs in *rh52a*, accounting for 4.4% of the embryos (Table 2).
(iii) Double mono-nucleate embryo sacs (DMES; Fig. 5Mb1–Mb3). A chalazal FM grew into a single mono-nucleate embryo sac in the WT (Fig. 5Wb). FMs developed into two respective mono-nucleate embryo sacs in *rh52a*. One was near the micropylar end, while the other was near the chalazal end.
(iv) Double bi-nucleate embryo sacs (DBES; Fig. 5Mc1–Mc3, Md3). In the WT, a mono-nucleate embryo sac subsequently underwent the first mitosis to become a bi-nucleate embryo sac in the WT (Fig. 5Wc), whereas in *rh52a* the this occurred in two mono-nucleate sacs, resulting in two bi-nucleate embryo sacs.
(v) Double tetra-nucleate embryo sacs (DTES; Fig. 5Md1, aMd2; Supplementary Fig. S8; Supplementary Videos S1, S2). A bi-nucleate embryo sac completed its second mitosis to generate a tetra-nucleate embryo sac in the WT (Fig. 5Wd), whereas in *rh52a* the second mitosis also occurred in two bi-nucleate embryo sacs, which separately formed two tetra-nucleate embryo sacs.
(vi) Double eight-nucleate embryo sacs (DEES; Fig. 6Ma1–Ma3; Supplementary Fig. S9; Supplementary Videos S3, S4). The third mitosis proceed in a tetra-nucleate embryo sac in the WT, leading to an eight-nucleate embryo sac (Fig. 5Wd). In *rh52a*, double tetra-nucleate embryo sacs also underwent their third mitosis to develop into DEES and finally differentiate into double-female-gametophytes (DFGs; two embryo sacs stacked in one ovule) (Fig. 6Mb1–Mb3).

**Table 2.** Frequencies of megasporogenesis and megagametogenesis observed in rice wild type plants and the two *rh52a* mutants

Genotype	No. ovaries examined	Type of megasporogenesis/megagametogenesis (%)						
		Multiple megasporocyte	Degenerated FM	DFM	DMES	DBES	DTES	DEES
Wild type	253	0.0	1.2	0.0	0.0	0.0	0.0	0.0
*rh52a-m1*	145	6.2	2.8	3.4	9.0	2.8	1.4	3.4
*rh52a-m2*	566	5.3	3.7	5.3	5.8	2.3	1.1	2.7
*rh52a* mean	–	5.8	3.3	4.4	7.4	2.6	1.3	3.1

FM, functional megaspore; DFM, double functional megaspore; ES, embryo sac; DMES, Double mono-nucleate ES; DBES, double bi-nucleate ES; DTES, double tetra-nucleate ES; DEES, double eight-nucleate ES.

**Table 3.** Frequencies of different types of observed embryo sacs in rice wild type plants and the two *rha52a* mutants

Genotype	No. ovaries examined	Type of embryo sac (%)								
		Normal fertilization ES	ESD	ESWF	ESWE	Stasis of fertilization ES	Zygotic fertilization ES	Non-fertilization ES	Fertilization DFG	Non-fertilization DFG
Wild type	207	93.2	2.4	0.1	2.9	1.4	0.5	1.4	0.0	0.0
*rh52a-m1*	290	46.6	6.6	7.9	2.1	1.0	3.4	15.5	4.1	12.4
*rh52a-m2*	257	34.6	8.2	12.5	5.1	0.7	0.8	17.1	2.7	13.6
*rh52a* mean	–	40.6	7.4	10.2	3.6	0.9	2.1	16.3	3.4	13.0
*rh52a* total*	–	–	–	49.5						

Combined results from 1 d and 3 d after fertilization are shown. ES, embryo sac; ESD, ES degeneration; ESWF, ES without female germ unit; ESWE, SE without egg apparatus; DFG, double-female-gametophyte (two embryo sacs stacked in one ovule).

* Total percentage of abnormalities across the two mutants excluding ESD.

Analysis of DFGs at megagametogenesis showed that in the *rh52a* mutants the double mono-nucleate embryo sacs represented 7.4%, bi-nucleates 2.6%, tetra-nucleate embryo sacs 1.3%, and eight-nucleate embryo sacs 3.1% (Table 2). Notably, asynchronous mitotic division was observed within DFGs during megagametogenesis (Figs 5Ma2, Mb2, Mb3, Mc2, Md2, 6Ma3; Supplementary Videos S2, S4). Embryo sacs at 1 DAF and 3 DAF were observed using WE-CLSM and abnormal fertilization was detected mainly in three types of embryo sacs in *rh52a* mutants, namely DFGs, embryo sacs without the egg apparatus, and embryo sacs without female reproductive units (Fig. 6). In total, abnormalities were observed in 49.5% of the ovaries examined in the mutants (Table 3). Among them, 3.4% of DFGs exhibited fertilization.

Notably, these fertilized DFGs showed that a single sac located towards the micropylar pole generated one embryo and free endosperm nuclei; however, the sac positioned near the chalazal pole did not exhibit this phenomenon (Fig. 6Mc1–Mc3). At 3 DAF, the initiation of endosperm cellularization occurred in the WT, producing the first layer of endosperm cells (Fig. 6Wd) however, despite fertilized embryo sacs generating free endosperm nuclei at 1 DAF in the *rh52a* mutants, they exhibited abnormal cellularization at 3 DAF (Fig. 6Md1).

### RH52A physically interacts with MFS1 and ZIP4

The complete coding sequence of *OsRH52A* was inserted into the pGBKT7 vector for yeast two-hybrid (Y2H) assays to identify specific interacting proteins. The full-length OsRH52A protein had no obvious toxicity and self-activation activity (Fig. 7A), so BD-OsRH52A was used as bait in our assays. Given that the RH52A protein is essential for FM development in rice, we hypothesized that it might directly interact with the proteins regulating embryo-sac development. Previous studies have shown that *MFS1* and *ZIP4* are embryo sac meiotic genes, and our Y2H assays indicated that OsRH52A interacted with the OsMFS1 and OsZIP4 proteins in yeast (Fig. 7B). These interactions were further confirmed *in vivo* via BiFC and luciferase complementation imaging (LCI) assays in tobacco leaf epidermal cells (Fig. 7C, D). YFP fluorescence signals were observed in the cytoplasm and nuclei of the tobacco cells for the fusion proteins of OsRH52A with both OsMFS1 and OsZIP4 (Fig. 7C).

### Knockout of rice *RH52A* affects the transcription of genes associated with embryo sac development

We next performed RNA-seq analysis on the embryo sac at the meiosis and FM stages for the WT and *rh52a-m2* genotypes. More than 251 million clean reads were detected in the WT and *rh52a-m2* ovaries during the two stages, and they were aligned against the Nipponbare reference genome, resulting in an average of 95.3% annotated transcripts of the reference genome in Q30 (Supplementary Table S3). Spearman correlation coefficients of the WT and *rh52a-m2* were between 0.68–0.97 among the three biological replicate samples (Supplementary Table S4). We selected 19 DEGs to validate the expression using qRT-PCR and correlation analysis demonstrated that their differential expression levels were consistent with the transcriptome data (Fig. 8A). All these results suggested that the transcriptome data were suitable for DEG screening and analysis.

Compared with WT, 39.1% (17438/44611) of the DEGs at meiosis and FM stage were detected in *rh52a-m2*, of which 8635 were up-regulated and 8803 were down-regulated (Supplementary Table S5). There were 1381 DEGs that were common to the two stages, of which 594 were up-regulated and 787 were down-regulated (Supplementary Tables S5, S6). GO analysis revealed that 787 down-regulated DEGs were significantly enriched in 43 GO terms in the biological processes category, including ‘microtubule-based movement’, ‘microtubule-based process’, ‘DNA conformation change’, and ‘cellular macromolecular complex assembly’ (Supplementary Table S7). Six GO terms were identified as significantly enriched in the cellular component category in the down-regulated DEGs, namely ‘nucleosome’, ‘protein-DNA complex’, ‘cell part’, ‘cell’, ‘external encapsulating structure’, and ‘cell wall’’, whilst 20 terms were significantly enriched in the molecular function category, including ‘microtubule motor activity’, ‘motor activity’, ‘hydrolase activity, acting on acid anhydrides, in phosphorus-containing anhydrides’ and ‘hydrolase activity, acting on acid anhydrides’’. In the up-regulated DEGs, 47 terms were significantly enriched in the biological processes category, including ‘oxidation reduction’, ‘response to biotic stimulus’, and ‘carbohydrate metabolic process’ (Supplementary Table S8). No terms were characterized in the cellular component category in the up-regulated DEGs, whilst a total of 32 terms were significantly enriched in the molecular function category, including ‘oxidoreductase activity’, ‘iron ion binding’, and ‘electron carrier activity’.

KEGG analysis identified 76 pathways for the down-regulated DEGs, of which the top five most enriched were ‘metabolic pathways’ (osa01100, accounting for 68 genes), ‘biosynthesis of secondary metabolites’ (osa01110, 40 genes), ‘starch and sucrose metabolism’ (osa00500, 14 genes), ‘motor proteins’ (osa04814, 13 genes), and ‘DNA replication’ (osa03030, 10 genes) (Supplementary Table S9). Mapping analysis showed that 17 down-regulated DEGs used ubiquitin-dependent systems as their protein degradation pathway (Fig. 8B; Supplementary Table S10). The KEGG analysis identified a total of 73 pathways for the up-regulated of which the top five most enriched were ‘metabolic pathways’ (osa01100, 65 genes), ‘biosynthesis of secondary metabolites’ (osa01110, 44 genes), ‘glycolysis/gluconeogenesis’ (osa00010, 14 genes), ‘carbon metabolism’ (osa01200, 14 genes), and ‘amino sugar and nucleotide sugar metabolism’ (osa00520, 10 genes) (Supplementary Table S11). There were three genes involved in the ‘ubiquitin mediated proteolysis’ pathway (osa04120).

The results of the GO and KEGG analyses indicated that the down-regulated DEGs had a significant role in embryo sac development. We therefore conducted a comparison between the 787 down-regulated DEGs that we observed and 5366 genes that have previously been identified as being expressed in rice reproductive organs (listed in Supplementary Table S2), and found that 234 of the 787 DEGs were associated with fertility (Supplementary Table S12). Importantly, two genes that are essential for embryo sac fertility were detected in the down-regulated DEGs, namely *MSP1* (*LOC_Os01g68870*) and *HSA1b* (*LOC_Os12g39920*) (Fig. 8C). Moreover, nine genes associated with embryo sac abortion were detected in the down-regulated DEGs during the FM stage, namely *ER2*, *Mre11*, *MEL1*, *PAIR3*, *APC6*, *CHR721*, *SHOC1*, *DES1*, and *PRD2* (Fig. 8C).

## Discussion

### *RH52A* plays an essential role in the female reproductive development of rice

The eukaryotic DEAD-box RNA helicase family proteins are essential for maintaining female gametophyte development during FM and mitosis (Huang *et al.*, 2010a; Chen *et al.*, 2020). Previous studies have shown that the Arabidopsis *sw3*,*rh36*, and *rh29* mutants have retarded progression of cell cycle during female gametophyte development, and *rh36* shows asynchronous female gametogenesis (Huang *et al.*, 2010a; Liu *et al.*, 2010; Chen *et al*., 2020). It has been demonstrated that *OsRH36* is able to restore the normal function of the *rh36* mutant allele in Arabidopsis, similar to the function of *AtRH36* (Huang *et al.*, 2010b). Here, we characterized the DEAD-box protein OsRH52A in rice. The OsRH52A protein was found to be localized to both the nucleus and cytoplasm (Fig. 1B, C), similar to the pattern of the OsRH36 and SWA3 (AtRH36) proteins being localized in the nucleus when the genes were expressed in onion epidermal cells (Huang *et al.*, 2010a, 2010b). Asynchronous development was identified in double-female-gametophytes during megagametogenesis (Figs 5Ma2, 5Mb3, 5Mc3, 5Md2, 6Ma3; Supplementary Videos S2, S4), with a phenotype identical to that observed in Arabidopsis *rh36*. The CRISPR/Cas9 mutations in *RH52A* caused disorders in female gametophyte development during gametogenesis, including multiple megasporocytes, degenerated FMs, double sets of female gametophytes, and defective mature embryo sacs (Figs 4–6; Tables 1, 2). These results indicated that *RH52A* plays an essential role in the female reproductive development of rice.

In rice, *MSP1* and *TDL1A* prevent the excessive entry of sporocytes into male and female sporogenesis, and their proteins interact with each other (Nonomura *et al.*, 2003; Zhao *et al.*, 2008). The defective ovules of the rice *er2* mutant show at least two AC-like cells or MMCs and excessive FMs during megasporogenesis, as well as two sets of female gametophytes within one ovule during megagametogenesis (Zhao *et al.*, 2020). Despite the identification of significant genes that govern the presence of additional female gametophytes in rice, the precise cytological mechanism underlying the occurrence of two sets of female gametophytes remains elusive. This lack of clarity can be attributed to the constraints imposed by the experimental instruments that are utilized to observe embryo sacs. In the present study, we were able to effectively examine the developmental phase of the DFG and its fertilization mechanism with the aid of WE-CLSM, a powerful cytological tool to observe embryo sac development. We found that the DFG developed from two nearby FMs in the same ovule, close to the chalazal portion (Fig. 8D). The two FMs underwent three mitotic divisions and formed two corresponding embryo sacs. Generally, one embryo sac of the DFG in the *rh52a* mutant, located at the micropylar end, harbored a female reproduction unit (including one egg, two synergids, and two polar nuclei) but no antipodal cells; the other had polar nuclei and antipodal cells but no egg apparatus (consisting of the egg cell and two synergids) (see Fig. 8D). Our results showed that the two embryo sacs in the DFG were different and that their specific positions determined the differentiation of egg apparatus and antipodal cells. As a consequence, the DFG varies in its fertilization: the embryo sac close to the micropylar end can fertilize and grow into an embryo and endosperm, while the other cannot since it has no egg cell and it is far from the micropylar end but close to the chalazal region. However, only 3.4% of DFGs could fertilize in the *rh52a* mutant, suggesting that they are different from normal embryo sacs in fertilization. In our study, ~16.3% of sterility in *rh52a* was caused by unsuccessful fertilization from a mature embryo sac even though it appeared to be structurally identical to a normal embryo sac (Table 3). Further study is required to elucidate the mechanism behind this intriguing finding.

During megagametogenesis, the embryo sac development of the spikelet is asynchronous and spans a long time. There is no strict positive correlation between the developmental stage of the embryo sac and spikelet length, making it difficult to accurately judge when to collect samples for examination, and hence potentially leading to unequal sample sizes at different stages. In this study, we found that we had sufficient numbers of embryo sacs during megasporogenesis and megagametogenesis to explain the developmental process of DFG (Table 2), and therefore we did not further expand the sample size. On the other hand, at the AC and FM stages an excessive number of archesporial-like and megaspore mother-like cells could be observed, but we were not able to observe their developmental track at the subsequent mitosis stage. Given the high proportion of degenerated mature sacs in *rh52a*, we presume that these cells degraded before entering the mono-nucleate stage.

### *RH52A* might affect the regulatory network of genes involved in functional megaspore development in rice

Our study demonstrated the interaction of rice RH52A with MFS1 and ZIP4 using Y2H, BiFC, and LCI assays (Fig. 7). *MFS1* and *ZIP4* are two key genes with different functions in the embryo sac during meiosis. MFS1, a coiled-coil protein, is indispensable for the repair of double-strand breaks, and its mutant exhibits abnormal FMs because of severe chromosome defects, resulting in a completely degenerated embryo sac before the mono-nucleate stage (J.Y. Lu *et al.*, 2020). ZIP4 plays an important role in crossover formation and homologous chromosome synapsis in meiosis, as female gametes from the *zip4* mutant are grossly impaired in these processes (Shen *et al.*, 2012); furthermore, *rh52a* had identical degraded or defective embryo sacs to those previously observed in the *zip4* and *mfs1* mutants (J.Y. Lu *et al.*, 2020). Therefore, the RH52A–MFS1 and RH52A–ZIP4 complexes are critical for the formation of FMs in rice.

Whilst the physical interactions between OsRH52A with both OsMFS1 and ZIP4 were demonstrated in this study (Fig. 7), female reproductive development is a complicated process in flowering plants and is governed by many genes and complex molecular mechanisms (Yang *et al.*, 2010; Erbasol Serbes *et al.*, 2019). RNA-seq is a potent method for studying gene expression and gene expression networks (Sun *et al.*, 2017), and in this study we found that many common down-regulated DEGs were enriched in the biological processes ‘microtubule-based movement’ and ‘microtubule-based process’ (Supplementary Table S7), which are required for female development or the maintenance and remodeling of embryo sac structure. Microtubules have dynamic characteristics of aggregation and depolymerization, and play an important role in maintaining cell morphology and in division processes (Wong and Hashimoto, 2017). In particular, microtubules are required to maintain the basic skeleton of the embryo sac, and the shape of the sac polar nuclei, egg apparatus, and antipodal cells within the embryo sac (Xu *et al.*, 1997) In addition, KEGG enrichment analysis showed 13 DEGs enriched in the ‘motor protein’ pathway (Supplementary Table S9). Motor proteins drive directional movement along microtubules or microfilaments by taking advantage of the energy released by ATP hydrolysis (Sweeney and Holzbaur, 2018). Motif III in DEAD-box proteins controls ATP hydrolysis (Cordin *et al.*, 2006), and our multiple amino acid sequence alignment of proteins from the DEAD-box RNA helicase family showed that RH52A possesses this motif (Fig. 1A). RH52A is therefore hypothesized to facilitate the provision of energy to motor proteins as they move along microtubules or microfilaments during ATP hydrolysis. The mutation in *RH52A* leads to the structure of the embryo sac being changed, which results in the embryo sac being aborted, and hence the microtubules within the sac are then degraded or remodeled. As a result of insufficient energy being provided by ATP hydrolysis, the genes related to microtubule assembly might be down-regulated, and/or genes involved in the motor protein pathway might also be down-regulated. Eleven DEGs associated with megasporogenesis or megagametogenesis were identified and confirmed to have lower expression levels throughout the meiosis and/or FM stages (Fig. 8C). Among them, *MSP1* and *HSA1b*, which are required for embryo sac development (Nonomura *et al.*, 2003; Kubo *et al.*, 2016), were detected in both the meiosis and FM stages. The other nine genes were identified at the FM stage, and they are involved in premeiotic mitosis, meiosis, or mitosis during embryo sac development (Nonomura *et al.*, 2007; Yuan *et al.*, 2009; Awasthi *et al.*, 2012; Ren *et al.*, 2019; Zhang *et al.*, 2020; Zhao *et al.*, 2020; Shen *et al.*, 2021; Hu *et al.*, 2023; Wang *et al.*, 2023).

In general, the observation of defective embryo sacs is preceded by damage to the molecular network mechanisms that regulate embryo sac development. Based on this, we speculate that rice *RH52A* is required for embryo sac development through the RH52A–MFS1 and RH52A–ZIP4 complexes that affect the expression level of crucial genes (*ER2*, *Mre11*, *MEL1*, *PAIR3*, *APC6*, *CHR721*, *SHOC1*, *DES1*, and *PRD2*), thereby regulating the development of the functional megaspore (Supplementary Fig. S10). Taken together, our study demonstrates the significance of meiosis and functional megaspores in the production of an eight-cell embryo sac.

## Supplementary data

The following supplementary data are available at *JXB* online.

Fig. S1. Phylogenetic tree of DEAD-box RNA helicase family proteins and a comparison of the coding sequence region between *OsRH52A*-2*x* and *OsRH52A*-4*x*.

Fig. S2. Phylogenetic tree of DEAD-box RNA helicase family proteins from different *Gramineae* species.

Fig. S3. Predicted protein–protein interactions for OsRH52A.

Fig. S4. Morphologic characteristics of the wild type and the *rh52a-m1* and *rh52a-m2* mutants.

Fig. S5. Images of plants and assessment of pollen fertility of the wild type and the *rh52a-m1* and *rh52a-m2* mutants.

Fig. S6. Pollen characteristics of the wild type and *rh52a-m1* and *rh52a-m2* mutants.

Fig. S7. Developmental stages of the normal embryo sac in the *rh52a-m2* mutant.

Fig. S8. Semi-thin sections of double tetra-nucleate embryo sacs in the *rh52a-m2* mutant.

Fig. S9. A series of 12 sections from the double eight-nucleate embryo sacs in the *rh52a-m2* mutant showing synchronous mitosis division.

Fig. S10. Functional model of OsRH52A regulating functional megaspore development.

Table S1. Primers used for qRT-PCR and plasmid construction.

Table S2. The 5366 genes previously reported for reproductive tissue expression in rice.

Table S3. Quality evaluation of RNA-seq data from ovaries of the wild type versus the *rh52a-m2* mutant at meiosis and the functional megaspore stage.

Table S4. Spearman correlation coefficients of between individual replicates of ovaries from the wild type and the *rh52a-m2* mutant.

Table S5. Numbers of DEGs from ovaries of the wild type versus the *rh52a-m2* at meiosis and functional megaspore stages.

Table S6. List of DEGs from ovaries of the wild type versus the *rh52a-m2* mutant at meiosis and functional megaspore stages.

Table S7. Gene ontology analysis of 787 common down-regulated DEGs from ovaries of the wild type versus he *rh52a-m2* mutant at meiosis and functional megaspore stages.

Table S8. Gene ontology analysis of 594 common up-regulated DEGs from ovaries of the wild type versus the *rh52a-m2* mutant at meiosis and functional megaspore stages.

Table S9. KEGG pathway enrichment analysis of 787 common down-regulated DEGs from ovaries of the wild type versus the *rh52a-m2* mutant at meiosis and functional megaspore stages.

Table S10. The 17 down-regulated DEGs mapped to ubiquitin degradation pathways in the *rh52a-m2* mutant.

Table S11. KEGG pathway enrichment analysis of 594 common up-regulated DEGs from ovaries of the wild type versus the *rh52a-m2* mutant at meiosis and functional megaspore stages.

Table S12. The 234 common down-regulated DEGs involved in meiosis and fertility in rice.

Video S1. The synchronous division of the double tetra-nucleate embryo sac of the *rh52a-m2* mutant.

Video S2. The asynchronous division of the double tetra-nucleate embryo sac of the *rh52*a-*m2* mutant.

Video S3. The synchronous division of the double eight-nucleate embryo sac of the *rh52a-m2* mutant.

Video S4. The asynchronous division of the double eight-nucleate embryo sac of the *rh52a-m2* mutant.

erae180_suppl_Supplementary_Figures_S1-S10_Video_legends

erae180_suppl_Supplementary_Tables_S1-S12

erae180_suppl_Supplementary_Video_S1

erae180_suppl_Supplementary_Video_S2

erae180_suppl_Supplementary_Video_S3

erae180_suppl_Supplementary_Video_S4

## Data Availability

The RNA-sequencing data are available at the NGDC (https://ngdc.cncb.ac.cn/) under the accession number PRJCA024426.
