# Supplementary material for: OsRH52A, a DEAD-box protein, regulates functional megaspore specification and is required for embryo sac development in rice
Source: J Exp Bot. 2024 Apr 20;75(16):4802–21. doi: 10.1093/jxb/erae180 (PMC11350083; doi:10.1093/jxb/erae180)
Supplement: erae180_suppl_Supplementary_Figures_S1-S10_Video_legends [file erae180_suppl_supplementary_figures_s1-s10_video_legends.pdf]

SUPPLEMENTARY DATA

Supplementary Video legends

Supplementary Video S1. The synchronous division of the double tetra-nucleate embryo sac of rh52a.

Supplementary Video S2. The asynchronous division of the double tetra-nucleate embryo sac of rh52a.

Supplementary Video S3. The synchronous division of the double eight-nucleate embryo sac of rh52a.

Supplementary Video S4. The asynchronous division of the double eight-nucleate embryo sac of rh52a.

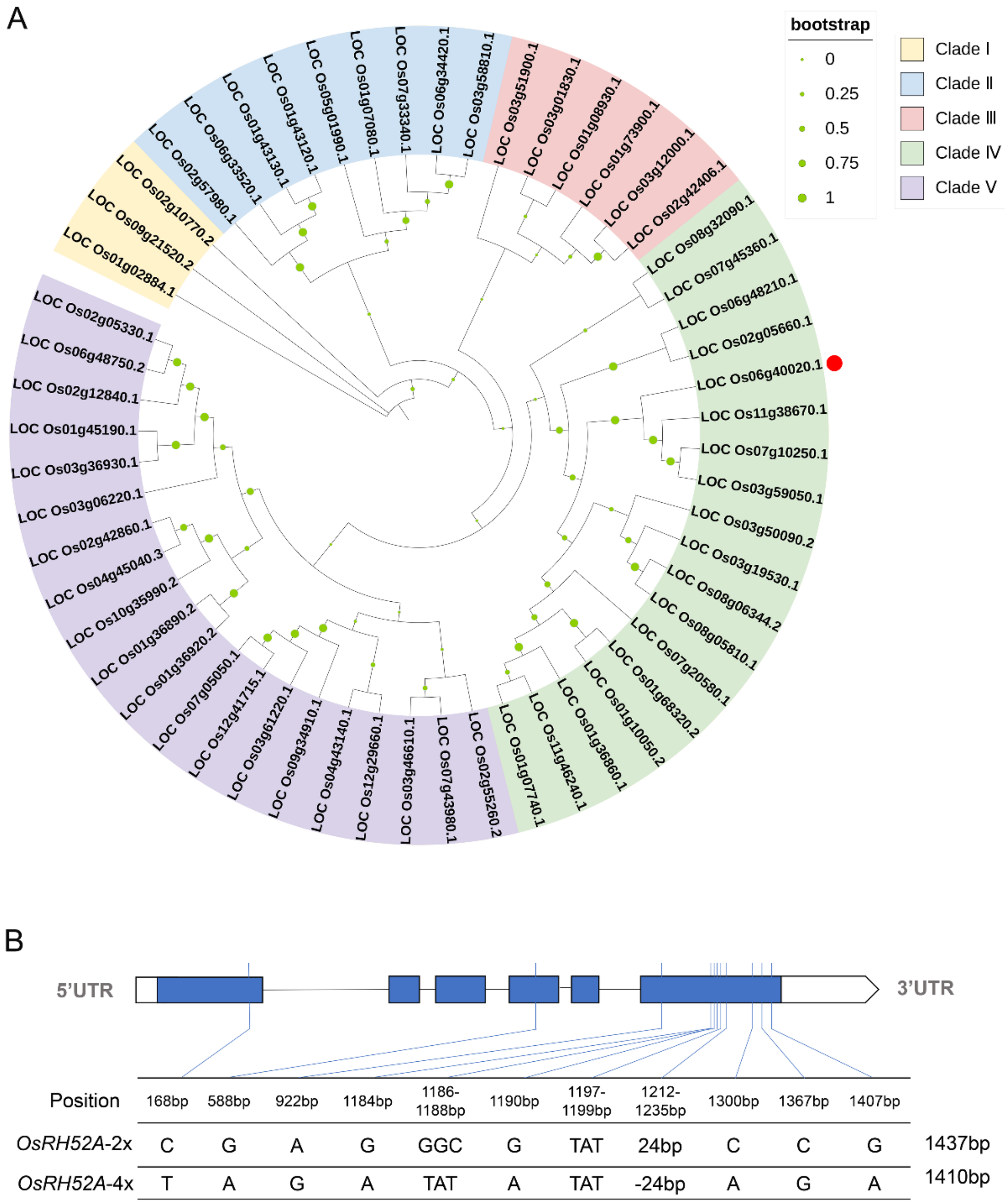

Supplementary Figure S1. Phylogenetic tree of DEAD-box family and the comparison of coding sequence region between *OsRH52A*-2x and *OsRH52A*-4x

A, Comparison of coding sequence region between OsRH52A-2x (diploid) and *OsRH52A*-4x（neo-tetraploid）based on whole genome sequencing. B, Phylogenetic tree analysis of 56 DEAD-box RNA helicase family members in rice. The size of the green dot on branches represents the bootstrap value. In rice, there are 56 genes belonging to the DEAD-box RNA helicase family, which are categorized into five clades. These clades are represented by the colors yellow, blue, red, green, and violet, corresponding to Clades I, II, III, IV, and V, respectively. The red circle denotes the gene *LOC_Os06g40020.1*, also known as *OsRH52A*.

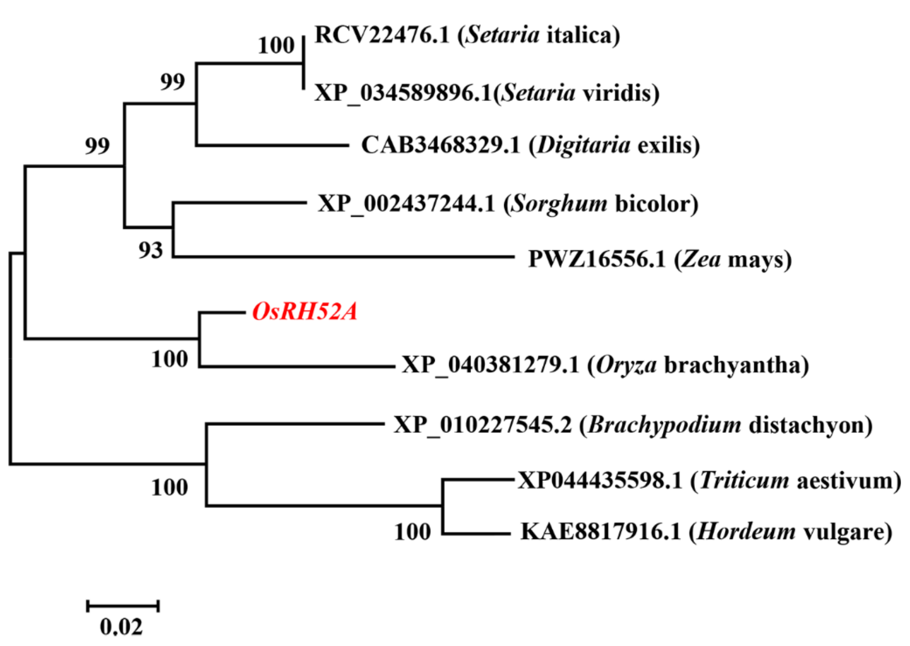

Supplementary Figure S2. Phylogenetic tree analysis of proteins from different species.

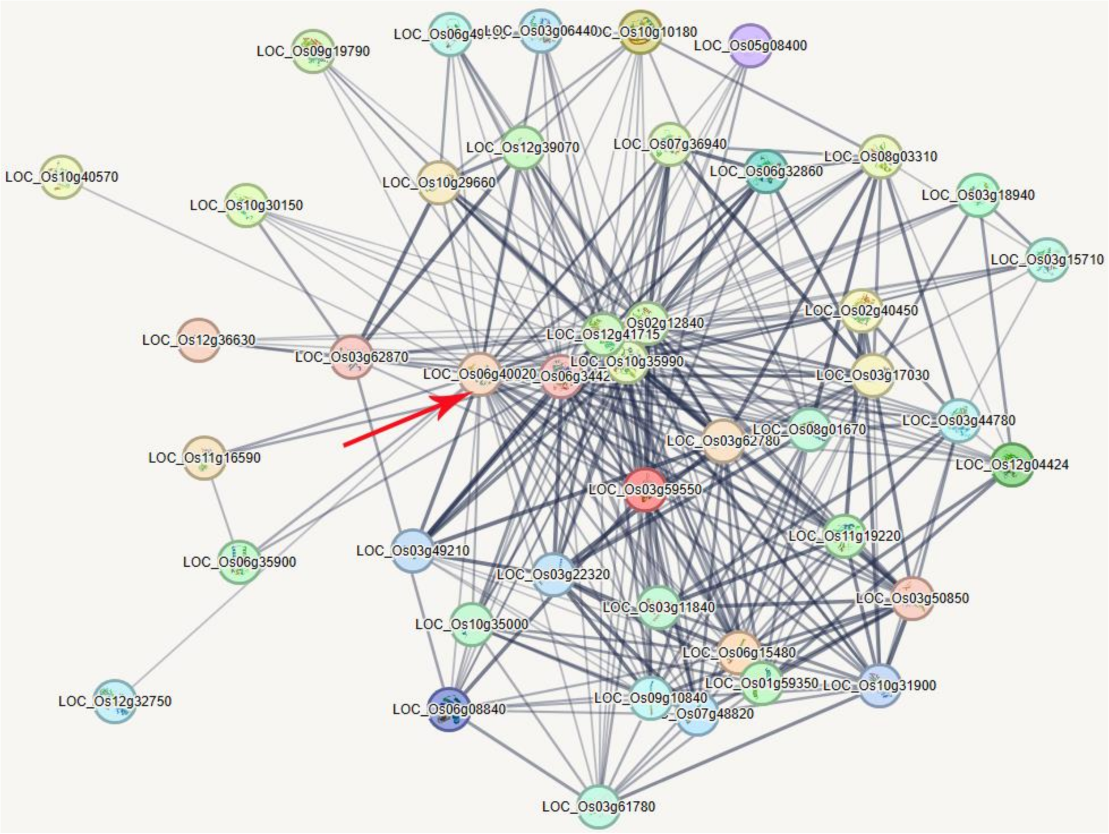

Supplementary Figure S3. Predicted protein-protein interaction for OsRH52A (LOC_Os06g40020)

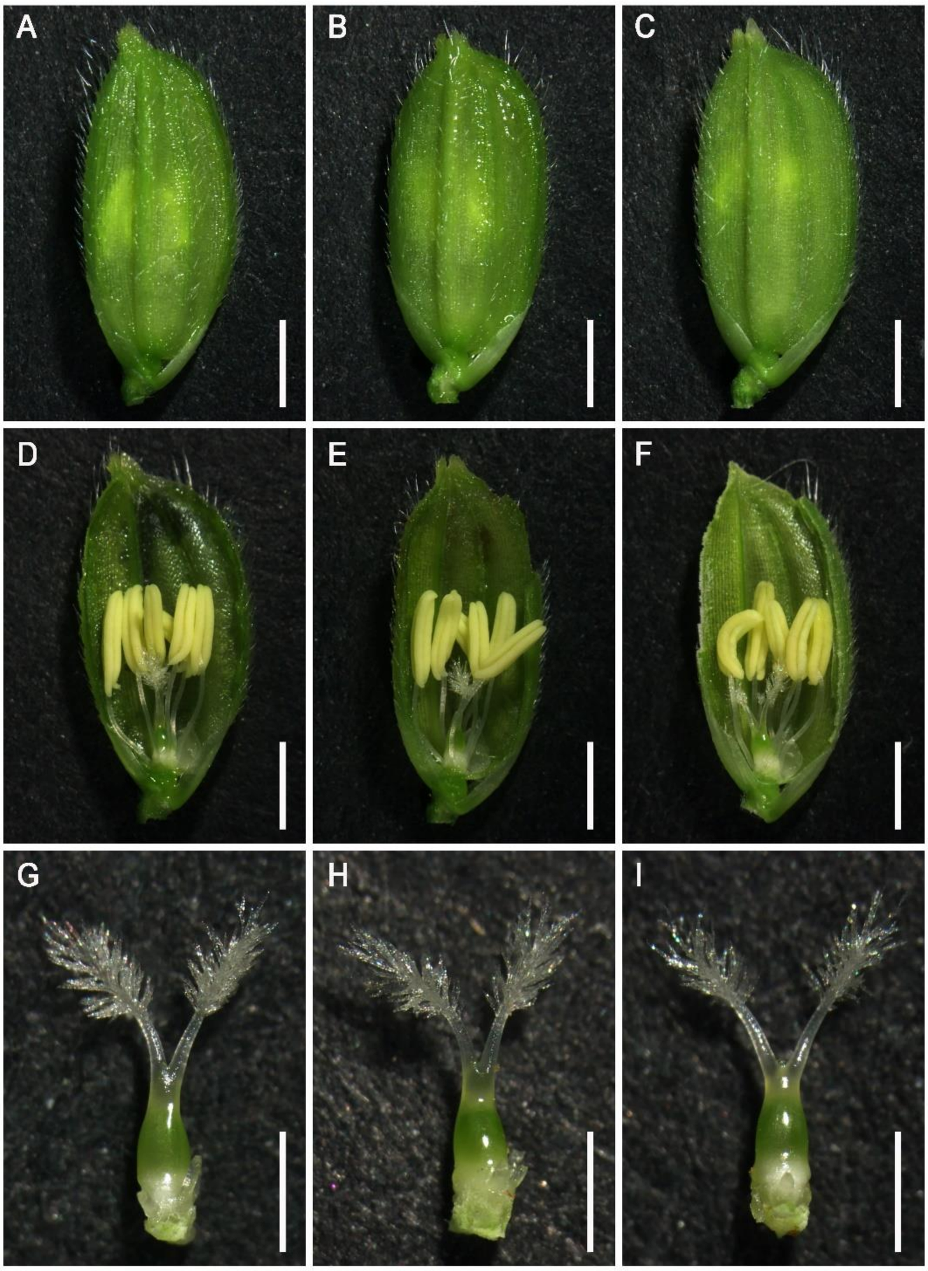

Supplementary Figure S4. Morphologic characteristics of WT, *rh52a-m1* and *rh52a-m2*.

A, D, and G indicate WT. B, E, and H indicate *rh52a-m1*. C, F, and I indicate *rh52a-m2*. Scale bars =20μm (A-F). Scale bars=10μm (G-I).

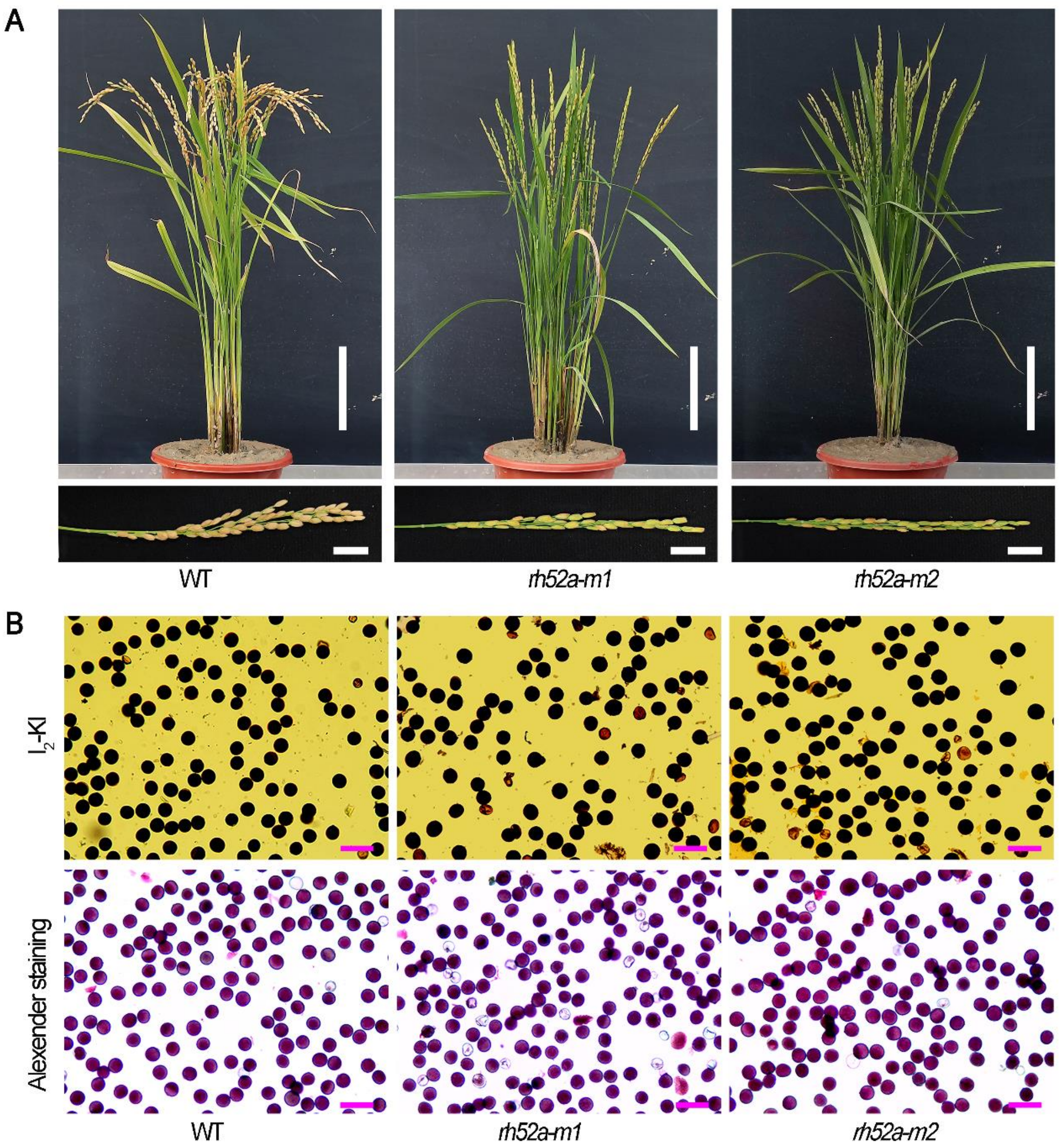

Supplementary Figure S5. Plants and pollen fertility of WT, *rh52a-m1*, and *rh52a-m2*. A, Plants and spikelets of WT, *rh52a-m1,* and *rh52a-m2*. Long scale bars=20cm, short scale bars=2cm. B, Pollen fertility of WT, *rh52a-m1,* and *rh52a-m2*. Scale bars=100μm.

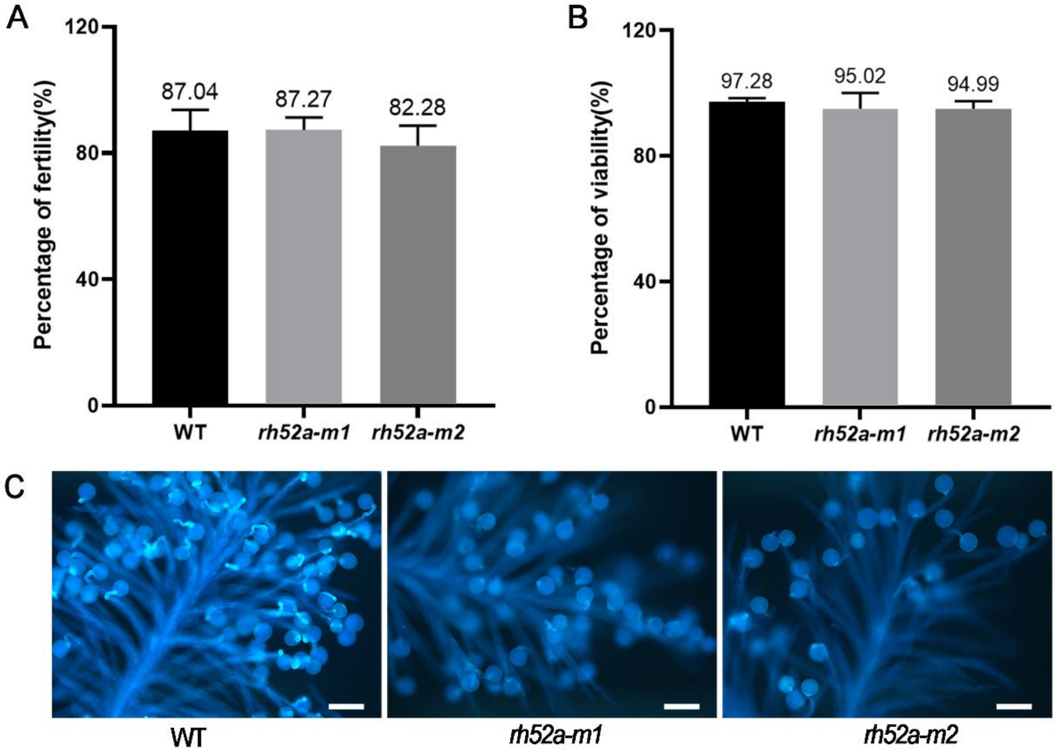

Supplementary Figure S6. Pollen characteristics of WT, *rh52a-m1,* and *rh52a-m2*.

A, Percentage of fertility in WT, *rh52a-m1,* and *rh52a-m2*. B, Percentage of viability in WT, *rh52a-m1,* and *rh52a-m2*. C, Pollen germination of WT, *rh52a-m1*, and *rh52a-m2* in vivo. Scale bars=100μm.

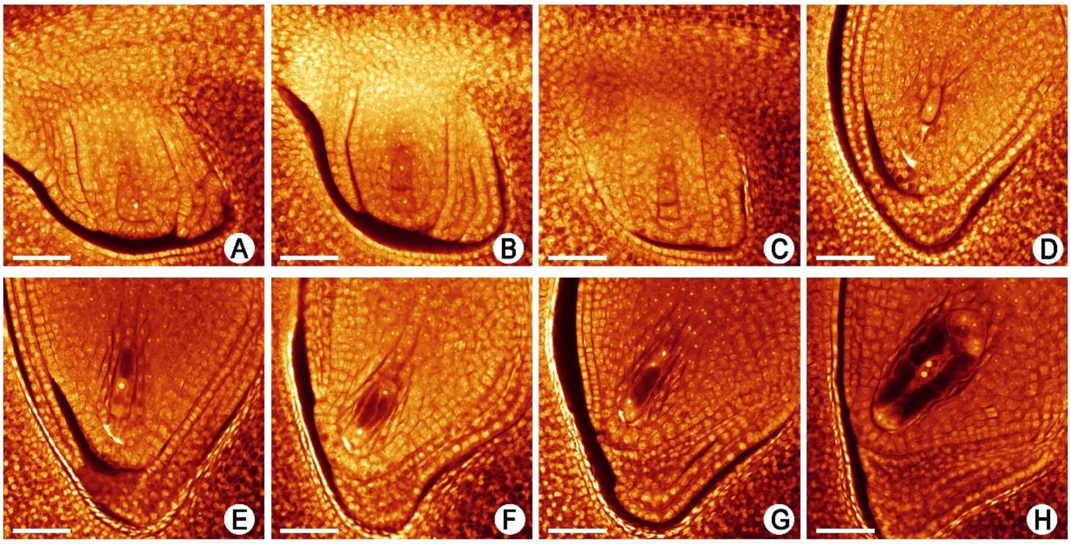

Supplementary Figure S7. Normal developmental stages of the embryo sac in *rh52a* mutants.

A, Megaspore mother cell (MMC). B, Dyad. C, Terad. D, Functional megaspore (FM). E, Mono-nucleate embryo sac. F, Bi-nucleate embryo sac. G, Tetra-nucleate embryo sac. H, Eight-nucleate embryo sac. Scale bars = 40μm.

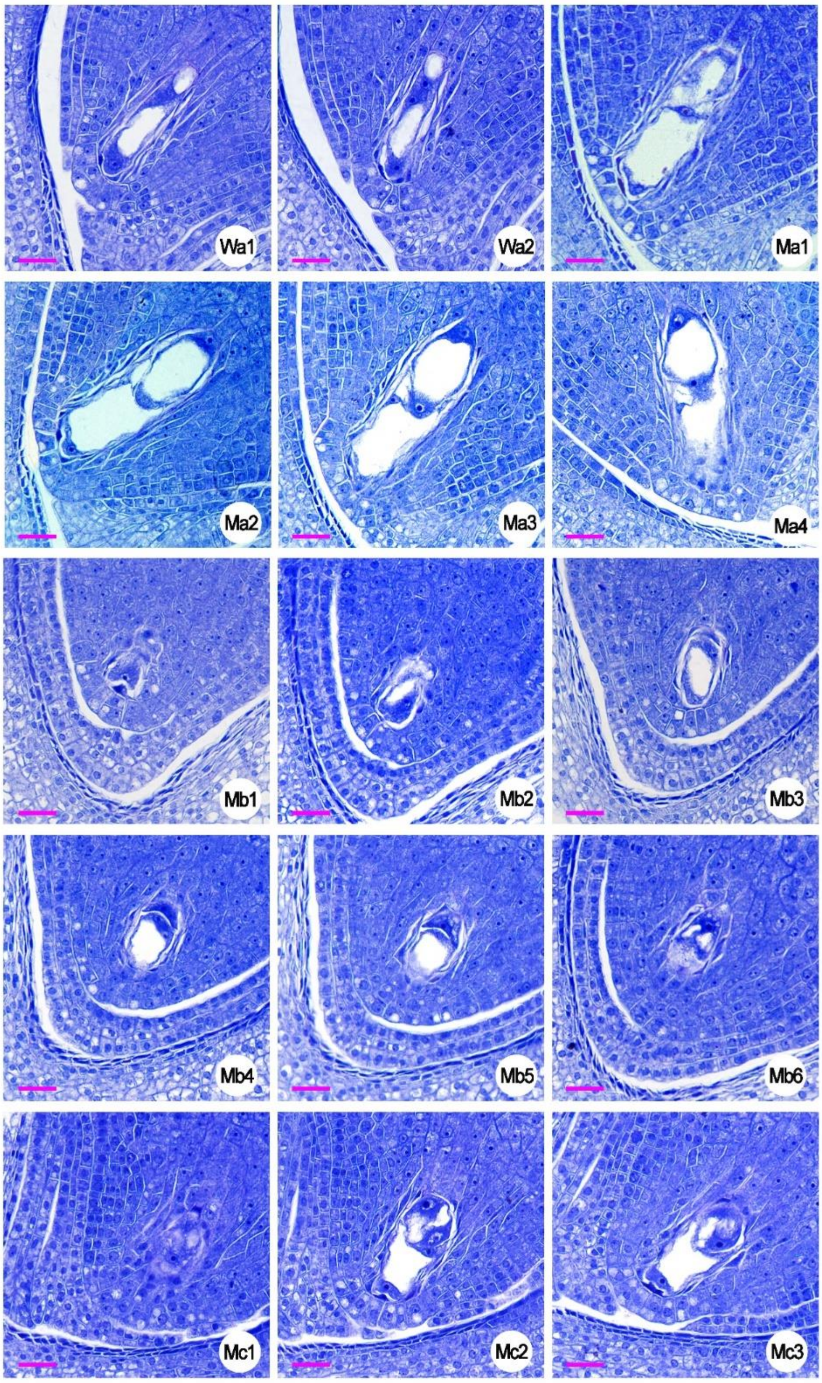

Supplementary Figure S8. Semi-thin sections of double tetra-nucleate embryo sacs in *rh52a* mutants.

Wa1 and Wa2, A series of two sections from a tetra-nucleate embryo sac in WT. Ma1 to Ma4, A series of four sections from a tetra-nucleate embryo sac in *rh52a* mutant (synchronous mitosis division). Mb1 to Mb6, A series of six sections from a tetra-nucleate embryo sac in *rh52a* mutant (synchronous mitosis division). Mc1 to Mc3, A series of six sections from a tetra-nucleate embryo sac in *rh52a* mutants (asynchronous mitosis division). Scale bars=20 μm.

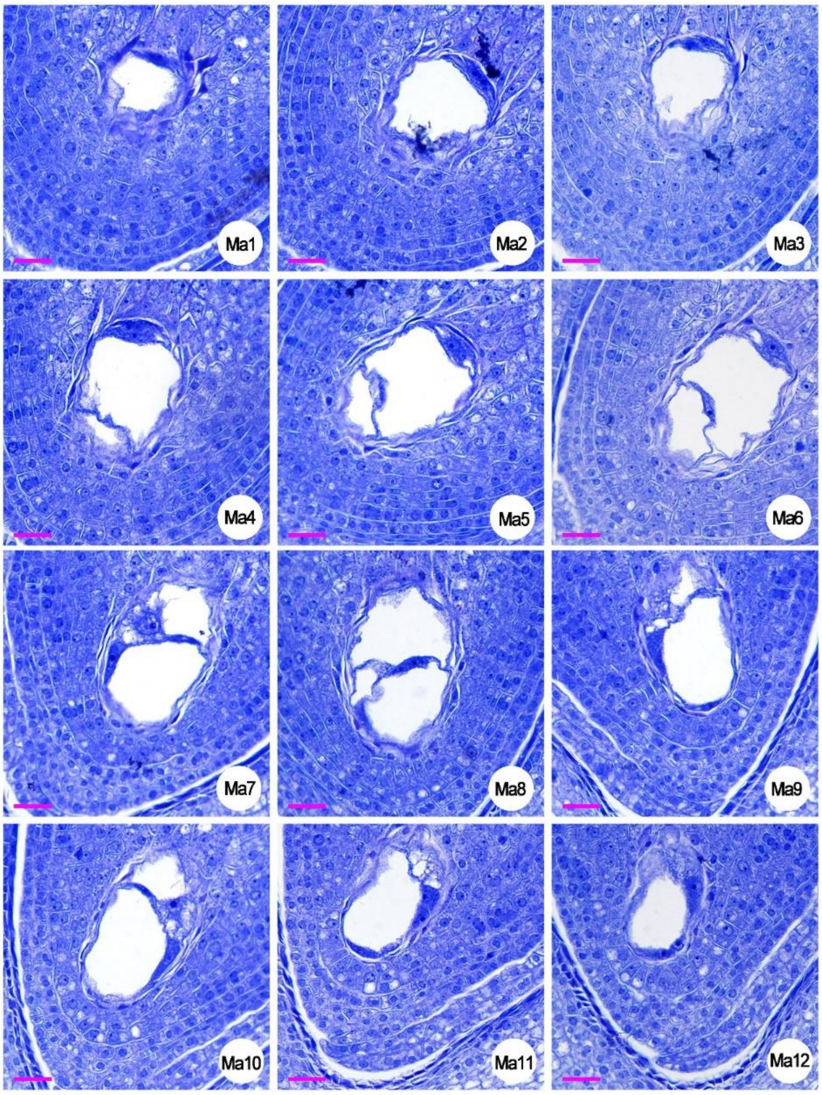

Supplementary Figure S9. A series of 12 sections from a double eight-nucleate embryo sac in *rh52a* mutants (synchronous mitosis division).

Scale bars=20 μm.

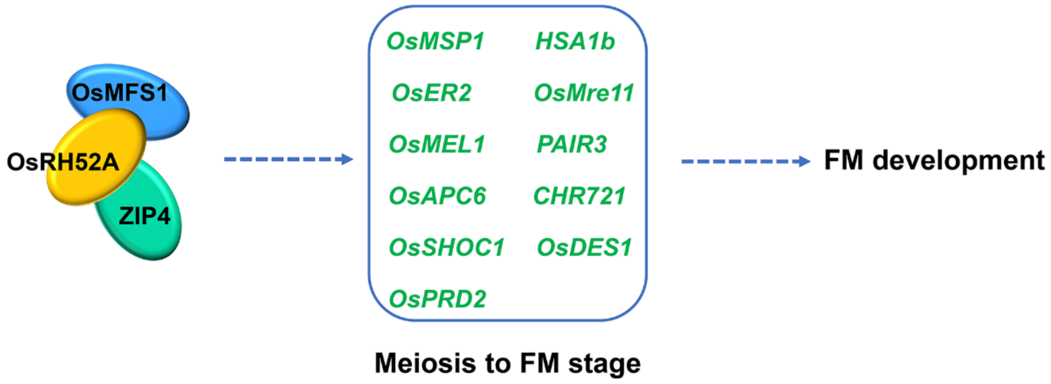

Supplementary Figure S10. Functional model of *OsRH52A* regulating FM development
